# Several posttranslational modifications act in concert to regulate gephyrin scaffolding and GABAergic transmission

**DOI:** 10.1038/ncomms13365

**Published:** 2016-11-07

**Authors:** Himanish Ghosh, Luca Auguadri, Sereina Battaglia, Zahra Simone Thirouin, Khaled Zemoura, Simon Messner, Mario A. Acuña, Hendrik Wildner, Gonzalo E. Yévenes, Andrea Dieter, Hiroshi Kawasaki, Michael O. Hottiger, Hanns Ulrich Zeilhofer, Jean-Marc Fritschy, Shiva K. Tyagarajan

**Affiliations:** 1 Institute of Pharmacology and Toxicology, University of Zurich, Winterthurerstrasse 190, CH 8057 Zurich, Switzerland; 2 Center for Neuroscience Zurich, CH 8057 Zurich, Switzerland; 3 Department of Molecular Mechanisms of Disease, University of Zurich, CH 8057 Zurich, Switzerland; 4 Department of Molecular and Systems Neurobiology, Graduate School of Medicine, The University of Tokyo, Tokyo 113-0033, Japan; 5 Institute of Pharmaceutical Sciences, Swiss Federal Institute of Technology, CH 8093 Zurich, Switzerland

## Abstract

GABA_A_ receptors (GABA_A_Rs) mediate the majority of fast inhibitory neurotransmission in the brain via synergistic association with the postsynaptic scaffolding protein gephyrin and its interaction partners. However, unlike their counterparts at glutamatergic synapses, gephyrin and its binding partners lack canonical protein interaction motifs; hence, the molecular basis for gephyrin scaffolding has remained unclear. In this study, we identify and characterize two new posttranslational modifications of gephyrin, SUMOylation and acetylation. We demonstrate that crosstalk between SUMOylation, acetylation and phosphorylation pathways regulates gephyrin scaffolding. Pharmacological intervention of SUMO pathway or transgenic expression of SUMOylation-deficient gephyrin variants rescued gephyrin clustering in CA1 or neocortical neurons of *Gabra2*-null mice, which otherwise lack gephyrin clusters, indicating that gephyrin SUMO modification is an essential determinant for scaffolding at GABAergic synapses. Together, our results demonstrate that concerted modifications on a protein scaffold by evolutionarily conserved yet functionally diverse signalling pathways facilitate GABAergic transmission.

## Abstract (web summary)

Gephyrin is a cytoplasmic scaffolding protein that selectively forms postsynaptic scaffolds at GABAergic and glycinergic synapses. Here the authors characterize regulatory mechanisms determining gephyrin scaffolding and GABAA receptor synaptic transmission that involve acetylation, SUMOylation and phosphorylation.

## Graphical abstract

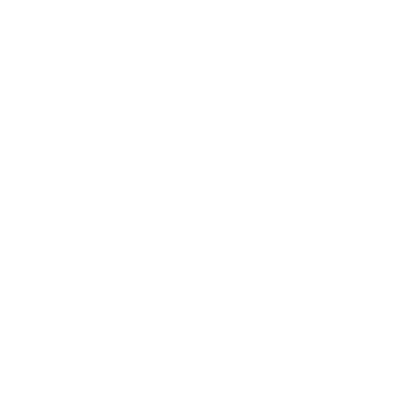

Small ubiquitin-like modifier (SUMO) conjugation to proteins is a widespread posttranslational modification (PTM) mechanism, which remarkably expands their structural and functional properties^1^,^2^,^3^. The SUMO conjugation pathway includes three SUMO isoforms (SUMO1/2/3); one E1 enzyme complex (SAE1/2); one E2 enzyme (UBC9); numerous E3 ligases, including members of the PIAS family (PIAS1, 2α, 2β, 3 and 4)^4^,^5^; and six deSUMOylating enzymes of the SENP family (SENP1, 2, 3, 5, 6, 7)^6^. Rapid deSUMOylation compared with SUMOylation can shift the equilibrium resulting in low steady-state level of SUMOylated proteins.

Although upstream signals strongly influence the ratio of SUMOylated versus deSUMOylated proteins, the downstream effects of SUMOylation usually involve altered interactions of modified proteins with other macromolecules, including proteins, DNA or RNA^6^, causing long-lasting changes in cellular function. Furthermore, cross-talk between different PTMs, such as phosphorylation-dependent SUMOylation or competition between SUMOylation and acetylation of the same lysine residues on specific substrates, expands this repertoire even further and allows rapid switches in protein functional states under the influence of a variety of signalling cascades^6^,^7^,^8^,^9^,^10^.

In neurons, SUMO conjugation of cytoplasmic and membrane proteins regulates many aspects of cell physiology and synaptic function in health and disease^11^,^12^. In particular, at glutamatergic synapses, SUMOylation of GluK2, Kv2.1, CASK and Arc influences both synapse formation and plasticity^12^,^13^. There is currently little knowledge about the occurrence and functional implications of SUMOylation at GABAergic synapses. In the current study, we identify gephyrin as a novel SUMO substrate and characterize the role of SUMOylation for modulating its functions *in vitro* and *in vivo*. Gephyrin is a cytoplasmic scaffolding protein that selectively forms postsynaptic scaffolds at GABAergic and glycinergic synapses, believed to anchor postsynaptic GABA_A_ receptors (GABA_A_R) and glycine receptors. In addition, the transsynaptic adhesion molecule neuroligin-2 might recruit gephyrin to inhibitory synapses via collybistin (CB) interactions^14^,^15^; however, the mechanistic basis underlying this recruitment process is still unclear. As a multifunctional protein, gephyrin is subject to extensive PTM by phosphorylation^16^, which was shown to be of immediate functional relevance. Thus, the phosphorylation status of residues S268 and S270 on gephyrin influences gephyrin scaffolding properties and thereby directly impinge on GABA_A_R synaptic function^17^,^18^.

On identifying specific gephyrin residues that are SUMO1- or SUMO2-conjugated *in vitro* and acetylated *in vivo*, we investigated possible crosstalk between SUMOylation, acetylation and phosphorylation of gephyrin and assessed the impact on the formation and size of gephyrin clusters in GABAergic synapses. Performing *in vivo* experiments in mice lacking the GABA_A_R α2 subunit, we could confirm the significance of gephyrin SUMOylation for its synaptic scaffolding function. Taken together, this study unravels the critical role for cellular signal transduction pathways in determining gephyrin SUMOylation, phosphorylation and acetylation at identified residues to regulate its scaffolding properties at specific postsynaptic sites and in turn regulating GABAergic function and plasticity.

## Results

### Gephyrin is a substrate for SUMOylation

**Figure 1:**
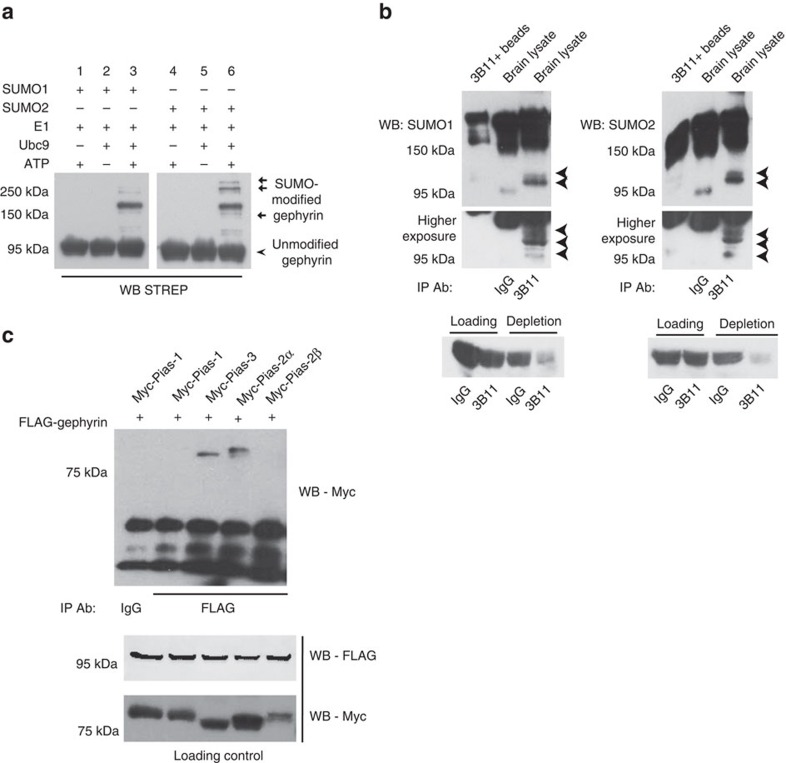
Biochemical assay demonstrating gephyrin is a SUMO substrate. (**a**) *In vitro* SUMO reaction using purified proteins show that gephyrin is a substrate for SUMO-1 and SUMO-2 conjugation, higher migrating gephyrin SUMO bands were detected using Strep-tactin horseradish peroxidase (HRP). (**b**) *In vivo* detection of gephyrin SUMO1 and SUMO2 conjugation. Arrowheads: SUMO modified gephyrin bands. Higher exposure of the SUMOylated gephyrin bands is shown below. Lower panel shows loading controls and depletion of gephyrin after IP specifically in 3B11 lanes (**c**) FLAG–gephyrin interaction with myc–PIAS3 and myc–PIAS2α in HEK293 cells.

SUMO conjugation is known to either facilitate or prevent inter- and intra-molecular interactions via conformation changes or direct steric hindrance. Currently, there are two online tools that are designed to predict SUMOylation sites in a protein sequence with different levels of confidence. Screening full-length rat gephyrin sequence in these online server-based algorithms (http://www.abgent.com/sumoplot; http://sumosp.biocuckoo.org/) identified several potential consensus SUMO motifs in gephyrin (Supplementary Table 1). To determine whether gephyrin can be SUMOylated, we expressed and purified from bacteria full-length STREP-gephyrin and other essential components for *in vitro* SUMO reaction, and performed SUMOylation assays^19^. Western blot (WB) analysis showed higher migrating SUMOylated bands of gephyrin, suggesting that it could be a novel substrate for SUMO1 and SUMO2 (Fig. 1a). The specificity of the reaction was established by excluding either Ubc9 or ATP from the reaction mix.

To validate these results *in vivo*, we immunoprecipitated (IP) gephyrin using the antibody 3B11 from C57Bl6/J mouse whole brain homogenate and performed WB using SUMO1 or SUMO2 antibodies, confirming gephyrin SUMOylation (Fig. 1b). We specifically identified SUMO1- and SUMO2-conjugated bands of gephyrin only in 3B11 lane.

The RING domain-containing SUMO E3 ligase PIAS proteins directly interact with their substrates for SUMOylation. Hence, we checked for gephyrin interaction with different PIAS family members. We co-expressed FLAG–gephyrin and myc–PIAS (1, 2α, 2β, 3) in HEK293 cells, IP'ed gephyrin using anti-FLAG antibody and performed WB for myc–PIAS. We could observe interaction between FLAG–gephyrin, and myc–PIAS3 and myc–PIAS2α (Fig. 1c), suggesting that these two PIAS isoforms might contribute to gephyrin SUMOylation.

### Identification of SUMO-conjugated Lys residues in gephyrin

**Figure 2:**
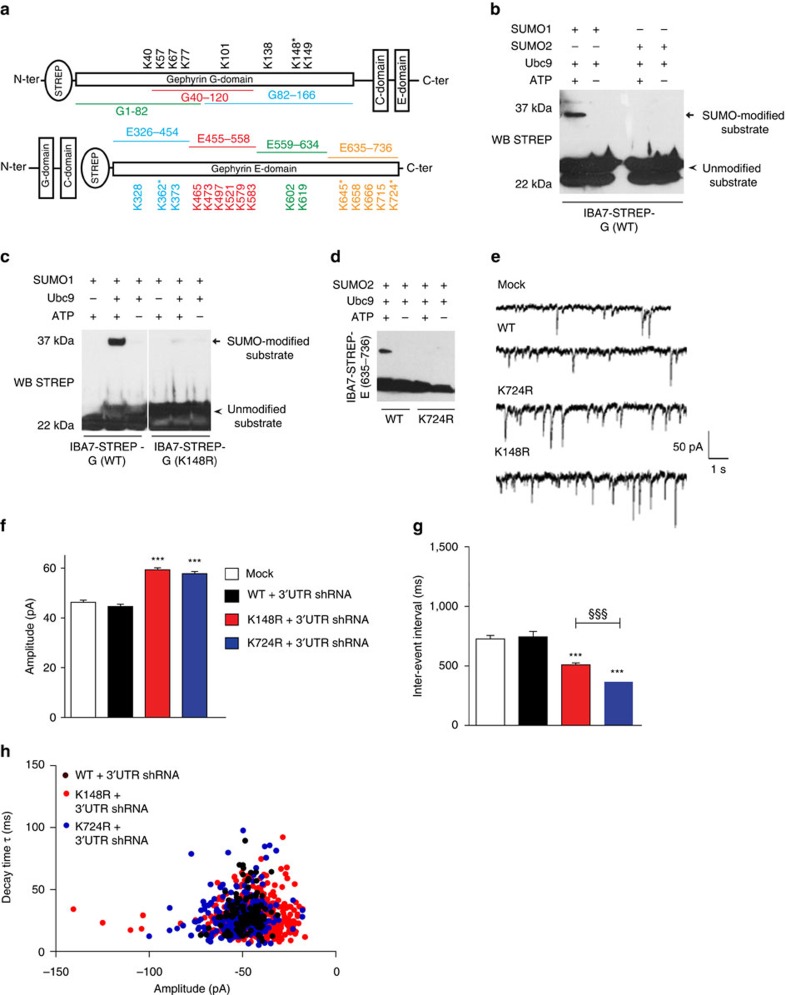
Identification and characterization of gephyrin SUMO sites. (**a**) Cartoon of gephyrin G-domain and E-domain peptide sequences with identified surface-exposed lysine residues. The identified SUMO1 and SUMO2 conjugation sites are marked *. (**b**) *In vitro* SUMOylation using bacterial expressed and purified STREP-G domain shows SUMO-1 conjugation but not SUMO2 conjugation. (**c**) *In vitro* SUMOylation using STREP-G (K148R) mutant abolishes SUMO-1 conjugation. (**d**) *In vitro* SUMO2 reactions using STREP-E (635–736) and STREP-E (635–736) K724R mutant shows SUMO2 conjugation in the WT peptide but SUMO2 conjugation is completely abolished in the K724R mutant peptide. (**e**) Sample mIPSC trace measurements in neurons transfected with eGFP–K148R, eGFP–K724R along with gephyrin 3′-UTR shRNA compared with WT or mock transfected neurons. (**f**) Average mIPSC amplitude in neurons transfected with eGFP–K148R, eGFP–K724R along with gephyrin 3′-UTR shRNA compared with WT or mock-transfected shows significantly increased amplitude. (**g**) Average mIPSC inter-event interval in neurons transfected with eGFP–K148R and eGFP–K724R along with gephyrin 3′-UTR shRNA compared with WT or mock-transfected shows significantly reduced inter-event intervals. (**h**) Decay kinetics of mIPSC currents in WT, K148R- and K724R-transfected neurons. Images from four independent experimental replicates were analysed; error bars are s.e.m.

Unstructured protein domains are preferred sites for phosphorylation, whereas structured domains are preferred sites for acetylation and SUMOylation^20^,^21^. As the central linker domain of gephyrin is highly unstructured and contains most of the phosphorylation sites identified so far, we focused on the structured G- and E-domains to identify putative SUMO sites. We screened for surface-exposed Lys residues on G- and E-domains based on crystal structure data^22^,^23^ (Fig. 2a and Supplementary Table 2). We observed that the majority of lysine residues are surface exposed on gephyrin. Therefore, we expressed G- and E-domains as smaller peptide fragments (80–100 amino acids) with an amino-terminal STREP-tag in bacteria and performed *in vitro* SUMO reactions. The first assay with purified STREP-G domain revealed a strong SUMO1-conjugated band, but no SUMO2 conjugation (Fig. 2b). To identify the SUMO1-conjugated residue on G-domain, we sequentially tested STREP-G1-82, STREP-G40-120 and STREP-G82-166 peptides for SUMO1 conjugation using *in vitro* assay (Supplementary Fig. 1a–c''). We also mutated surface-exposed lysine residues in each of the peptides and tested them for *in vitro* SUMO1 conjugation. The K148R point mutation in peptide STREP-G82-166 abolished SUMO1 conjugation (Supplementary Fig. 1c'). To confirm K148 as an authentic SUMO1 site, we introduced K148R mutation into the whole length STREP-G domain and performed *in vitro* SUMO reaction (Fig. 2c). Mutation of residue K148 abolished G-domain SUMO1 conjugation.

Similarly, we tested for SUMO1 and SUMO2 conjugation on the gephyrin E-domain using bacterially purified STREP-E domain. *In vitro* SUMO assays showed both SUMO1 and SUMO2 conjugation on the gephyrin E domain. To identify specific SUMO sites, we tested E domain peptide fragments (wild type (WT) or mutant) in the *in vitro* assay (Supplementary Fig. 1e–h). We identified K326 and K645 as additional SUMO1 sites on gephyrin E domain. However, we only found one peptide, STREP-E635-736, with SUMO2 conjugation. Hence, we mutated the surface-exposed lysine K724 and confirmed it as a SUMO2 conjugation site (Fig. 2d).

Based on these results, we chose to focus on K148 (SUMO1) site and K724 (SUMO2) site for detailed characterization. These two residues are also close to the G-domain dimer and E-domain trimer interface, making them prabable candidates for gephyrin scaffold regulation. Hence, we assessed the effects of abolishing SUMO conjugation of these two residues on inhibitory neurotransmission by measuring GABAergic mini inhibitory postsynaptic currents (mIPSC) amplitude and inter-event intervals. To this end, we co-transfected cultured hippocampal neurons with eGFP–gephyrin, eGFP–K148R or eGFP–K724R, along with gephyrin 3′-untranslated region (3′-UTR) short hairpin RNA (shRNA)^24^, to avoid a possible influence of endogenous gephyrin on the phenotype of eGFP–gephyrin SUMO1 and SUMO2 mutants (Supplementary Fig. 2a–d). Cells were transfected at 8 DIV and recorded mIPSC from 6 days later (8+6 DIV), using mock-transfected cells as control (Fig. 2e). Neurons expressing SUMO1-deficient eGFP–K148R mutant and SUMO2-deficient eGFP–K724R mutant had larger mIPSC amplitudes and shorter inter-event intervals compared with WT eGFP–gephyrin (Fig. 2f–g and Supplementary Table 3), suggesting that preventing gephyrin SUMOylation might enhance its postsynaptic clustering along with GABA_A_Rs and facilitate GABAergic synaptic transmission. Interestingly, the SUMO2 site mutant exhibited a more pronounced decrease in mIPSC inter-event intervals (Supplementary Fig. 2f and Supplementary Table 3).

The decay kinetics of mIPSCs in neurons transfected with eGFP–gephyrin, eGFP–K148R or eGFP–K724R were not significantly different (Fig. 2h). It is possible that gephyrin SUMOylation indiscriminately affects the majority of gephyrin-containing GABAergic synapses.

### PIAS3 influences gephyrin postsynaptic clustering

We tried to identify the underlying mechanism for gephyrin SUMOylation next. Many synaptic proteins have been identified as SUMO substrates *in vivo*^25^. Hence, we tested whether SUMO1 and SUMO2 are co-localized with eGFP–gephyrin at GABAergic postsynaptic sites in primary hippocampal neurons co-transfected (DIV8) with eGFP–gephyrin and myc–SUMO1 or myc–SUMO2. We stained for myc and VGAT at DIV15 and analysed for co-labelling using laser confocal microscopy (Supplementary Fig. 3a–b''). We found three distinct subcellular localizations of myc–SUMO1 and myc–SUMO2 in primary neurons; namely, only nuclear, only cytosolic and both nuclear and cytosolic. However, in neurons exhibiting dendritic distribution of myc–SUMO1 or myc–SUMO2, the staining was co-localized with eGFP–gephyrin clusters apposed to VGAT-positive terminals (Supplementary Fig. 3a–b'', lower panels). Subcellular localization differences in neurons have not been reported earlier; we confirmed our observations using specific antibodies against endogenous SUMO1 and SUMO2 (Supplementary Fig. 3c–d'). Similar to myc–SUMO1/2 overexpression, antibody staining for endogenous SUMO1 and SUMO2 also revealed subcellular localization differences within the nucleus, soma and dendrites, suggesting that they are not due to protein overexpression artefacts.

**Figure 3:**
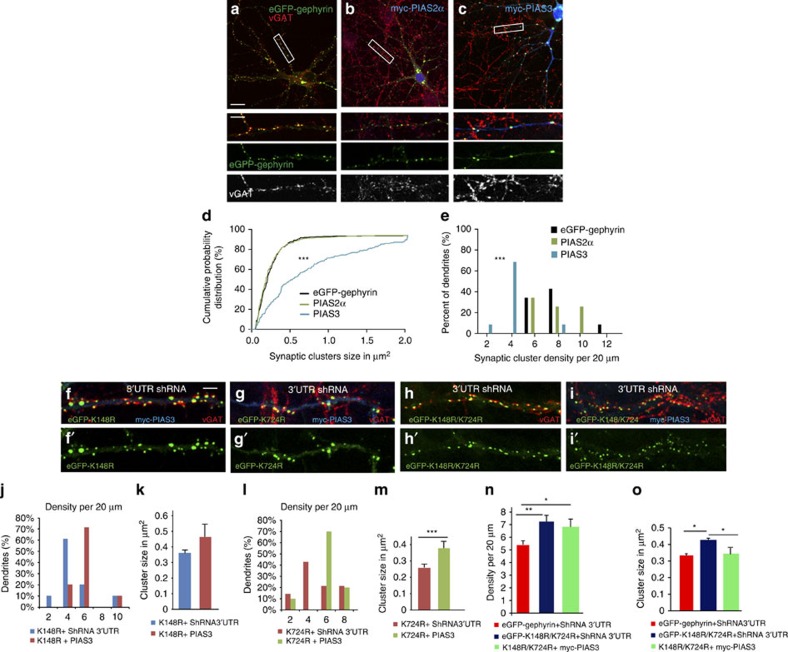
PIAS affect eGFP–gephyrin clustering in primary hippocampal neurons. (**a**–**c**) Primary hippocampal neurons co-transfected with eGFP–gephyrin and myc–PIAS2α or myc–PIAS3 (DIV 8+7) shows morphological differences in the presence of myc–PIAS3. (**d**) Cumulative probability distribution of eGFP–gephyrin clusters shows significant increase in cluster size in neurons expressing myc–PIAS3. (**e**) Synaptic cluster density distribution of eGFP–gephyrin in neurons co-transfected with myc–PIAS shows significant reduction in neurons expressing myc–PIAS3. (**f**–**i'**) Morphology of neurons transfected with eGFP–K148R, eGFP–K724R or eGFP–K148R/724R and 3′-UTR shRNA with or without myc–PIAS3. (**j**) PIAS3 co-expression significantly increases the cluster density in eGFP–K148R neurons. (**k**) Mean cluster size is not altered in eGFP–K148R mutant with myc–PIAS3 co-expression. (**l**) Myc–PIAS3 co-expression does not affect cluster density in K724R-expressing neurons. (**m**) Mean cluster size is significantly increased in K724R with the co-expression of myc–PIAS3. (**n**) SUMO double-mutant eGFP–K148R/K724R has significantly higher mean cluster density compared with eGFP–gephyrin. (**o**) Mean cluster size of eGFP–K148R/K724R is also significantly bigger compared with eGFP–gephyrin. Myc–PIAS3 co-expression does not affect cluster density in eGFP–K148R/K724R-expressing neurons. Mean cluster size of eGFP–K148R/K724R returns to eGFP–gephyrin control levels with the co-expression of myc–PIAS3. Scale bars, 10 μm and 5 μm. Images from five independent experimental replicates were analysed; error bars are s.e.m.

To influence gephyrin SUMOylation levels in primary neurons, we co-expressed myc–PIAS2α or myc–PIAS3 along with eGFP–gephyrin, as these two PIAS family members specifically interact with gephyrin (Fig. 1c). We analysed DIV8+7 neurons co-transfected with PIAS2α or PIAS3 for changes in eGFP–gephyrin cluster size or density (Fig. 3a–e). Image quantification showed that myc–PIAS2α co-expression does not change eGFP–gephyrin clustering compared with eGFP–gephyrin-alone control (Fig. 3d,e). However, neurons expressing myc–PIAS3 had larger and fewer eGFP–gephyrin clusters at GABAergic synapses (Fig. 3c, lower panels), as determined by their apposition to VGAT-positive terminals. Quantitative analysis (Fig. 3d) showed that myc–PIAS3 co-expression significantly increased eGFP–gephyrin cluster size (0.8±0.04 versus 0.2±0.01 μm^2^; one-way analysis of variance (ANOVA) *F*_(2, 1,786)_=61.45, Bonferonni *post-hoc* test, *P*<0.0001), while reducing their density (3.2±0.05 per 20 μm dendrite segment versus 6.5±0.6; one-way ANOVA *F*_(2, 48)_=4.2, Bonferonni *post-hoc* test, *P*<0.05).

It is well established that substrate specificity for SUMO conjugation and de-conjugation is enhanced in the presence of specific PIAS family members via their direct interaction with the substrate. Having identified K148 and K724 as SUMO1 and SUMO2 sites on gephyrin, we tested for PIAS3 effect on gephyrin SUMO mutations. We co-expressed myc–PIAS3 along with eGFP–K148R or eGFP–K724R in cultured neurons (Fig. 3f,g'). Analysis at DIV 8+7 revealed clear phenotypic differences between K724R and K148R mutants in the presence of PIAS3 (Fig. 3j–m). Co-expression of myc–PIAS3 with eGFP–K148R increased the cluster density (5±0.5 versus 3.6±0.2, Mann–Whitney test, *P*=0.02; Fig. 3j), whereas not affecting the cluster size (0.45±0.08 versus 0.36±0.1 μm^2^, Mann–Whitney test, *P*=0.26; Fig. 3k). Conversely, cluster density in neurons expressing eGFP–K724R and myc–PIAS3 was not altered (4.2±0.4 versus 5.2±0.5; Mann–Whitney test, *P*=0.57; Fig. 3l), but their size was significantly increased (0.25±0.01 versus 0.37±0.01 μm^2^; Mann–Whitney test, *P*<0.0001; Fig. 3m).

To examine the simultaneous influence of SUMO1 and SUMO2 on gephyrin, we generated eGFP–K148R/K724R double mutant and transfected hippocampal primary neurons in the presence of gephyrin 3′-UTR shRNA (Fig. 3h,h'). Quantitative analysis showed significant increase in eGFP–K148R/K724R cluster density compared with eGFP–gephyrin (7.6±0.5 versus 5±0.3; one-way ANOVA *F*_(2, 31)_=7.8, Bonferonni *post-hoc* test, *P*=0.0017; Fig. 3n), as well as size (0.41±0.01 versus 0.32±0.01 μm^2^; one-way ANOVA *F*_(2, 1,223)_=4.8, Bonferonni *post-hoc* test, *P*<0.0001; Fig. 3o). If K148 and K724 are authentic SUMO1 and SUMO2 sites that regulate gephyrin scaffolding, the double mutant eGFP–K148R/K724R should be insensitive to myc–PIAS3 expression. To test this, we transfected eGFP–K148R/K724R, gephyrin 3′-UTR shRNA and myc–PIAS3 into primary neurons and analysed for clustering change at DIV 8+7 (Fig. 3i,i'). Analysis for cluster density showed no change for eGFP–K148R/K724R double mutant in the presence or absence of myc–PIAS3 (Fig. 3n). Interestingly, the cluster size of eGFP–K148R/K724R in the presence of myc–PIAS3 returned to WT eGFP–gephyrin baseline levels (0.41±0.01 versus 0.32±0.01, one-way ANOVA *F*_(2, 1,223)_=4.8, Bonferonni *post-hoc* test, *P*<0.0001, Fig. 3o). These observations suggested for additional regulatory mechanism that could influence PIAS3 SUMO conjugation on gephyrin at K148R and K724R.

### PIAS3 gephyrin regulation is phosphorylation dependent

**Figure 4:**
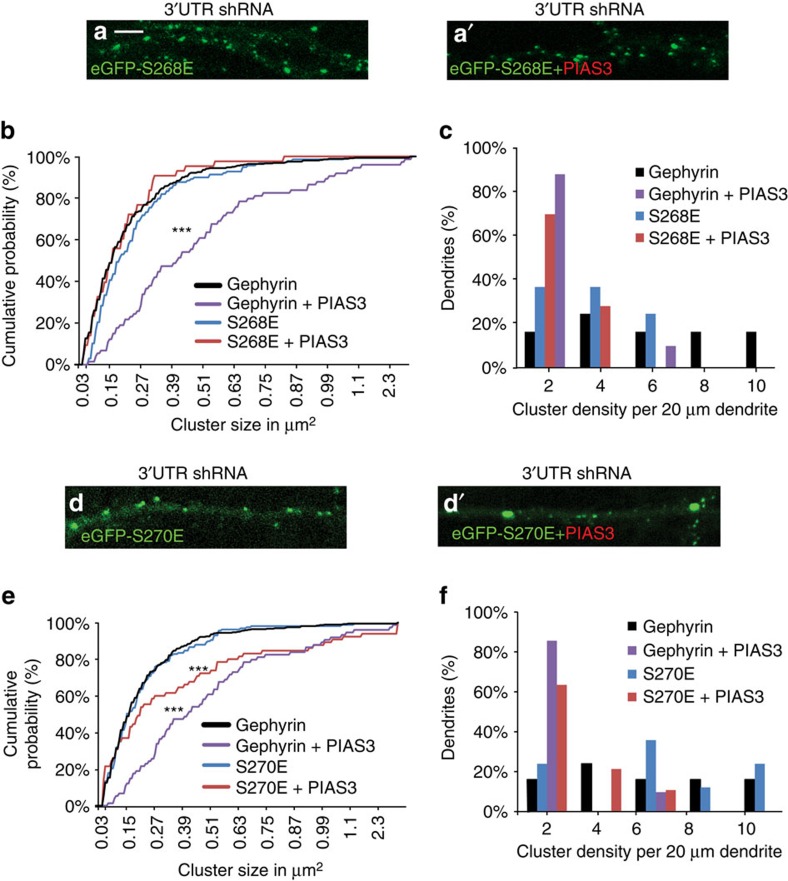
Morphology of ERK1/2 phosphorylation mimicking eGFP–S268E gephyrin mutant in neurons co-transfected with myc–PIAS3 (DIV 8+7). (**a**,**b**) Cumulative probability distribution of eGFP clusters in the presence of myc–PIAS3 shows that phosphorylation at S268 blocks PIAS3-mediated cluster growth. (**c**) Synaptic cluster density distribution in neurons co-expressing myc–PIAS3 shows that gephyrin phosphorylation at S268 cannot prevent PIAS3-mediated reduction in cluster density. (**d**,**d'**) Morphology of GSK3β phosphorylation mimicking eGFP–S270E gephyrin mutant in neurons co-transfected with myc–PIAS3 (DIV 8+7). (**e**) Cumulative probability distribution of eGFP clusters in neurons expressing myc–PIAS3 shows that phosphorylation at S270 does not block PIAS3-mediated size growth. (**f**) Synaptic cluster density distribution in neurons co-transfected with myc–PIAS3 shows significant reduction compared to eGFP–S270E alone control cells. Scale bar, 5 μm. Images from three independent experimental replicates were analysed; error bars are s.e.m.

It has been reported that phosphorylation at S268 by ERK1/2 regulates the size and phosphorylation at S270 by GSK3β regulates the density of postsynaptic gephyrin clusters^17^,^18^. We wondered whether gephyrin phosphorylation at S268 and S270 would influence the effect of PIAS3 on gephyrin clustering. Therefore, we co-transfected myc–PIAS3 and eGFP–S268E mutant (charged residue mimicking constitutive phosphorylation) along with gephyrin 3′-UTR shRNA and analysed for changes in eGFP–S268E clustering in DIV 8+7 neurons (Fig. 4a,a'). The co-expression of myc–PIAS3 did not increase the cluster size of eGFP–S268E mutant, unlike WT eGFP–gephyrin (Fig. 4b; 0.3±0.01 μm^2^ versus 0.25±0.01 μm^2^; one-way ANOVA *F*_(4,756)_=7.5, *P*<0.0001, Bonferonni *post-hoc* test, not significant). Hence, phosphorylation status at S268 influences PIAS3-dependent changes in gephyrin cluster size. However, when we compared cluster density, we found that myc–PIAS3 co-expression significantly reduced eGFP–S268E cluster density (3.5±0.5 versus 1.6±0.4; one-way ANOVA *F*_(4,39)_=6.5, *P*=0.0004; Fig. 4c), similar to PIAS3 effect on WT.

Next, we analysed the possible contribution of S270 phosphorylation status on myc–PIAS3-dependent increase in cluster size and reduction in cluster density. We analysed DIV 8+7 neurons co-transfected with eGFP–S270E and gephyrin 3′-UTR shRNA, or with eGFP–S270E, myc–PIAS3 and gephyrin 3′-UTR shRNA (Fig. 4d,d'). Quantitative analysis of eGFP–S270E revealed a significant increase in cluster size in the presence of myc–PIAS3 (0.55±0.07 versus 0.32±0.01 μm^2^; Fig. 4e; one-way ANOVA *F*_(4,756)_=7.5, *P*<0.0001), confirming that S268 residue is specifically involved in regulation of cluster size. Interestingly, expression of eGFP–S270E did not prevent the myc–PIAS3-induced reduction in gephyrin cluster density (2.0±0.4 versus 5.5±1.06; one-way ANOVA *F*_(4, 39)_=6.5, *P*=0.0004; Fig. 4f), indicating that PIAS3 can override the effects of S270 phosphorylation-dependent modulation of gephyrin cluster density. These results reveal a possible crosstalk between the PIAS3 and ERK1/2 kinase pathways for gephyrin scaffold size regulation.

### Gephyrin is acetylated at K666

**Figure 5:**
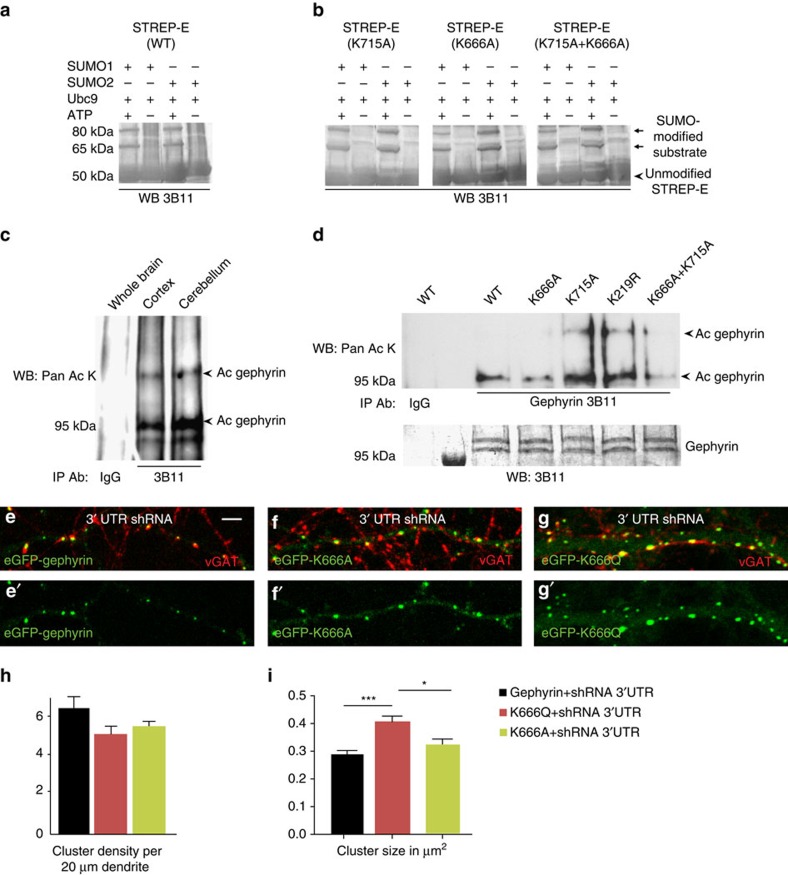
K666 is a novel acetylation site on gephyrin. (**a**) *In vitro* SUMO reaction to using bacterial expressed and purified STREP-E domain shows both SUMO1 and SUMO2 conjugation at the carboxy terminus. (**b**) *In vitro* SUMO reaction using consensus site mutants K666A, K715A and K666A+K715A shows SUMOylation. (**c**) *In vivo* analysis after denaturing IP for gephyrin and WB for Pan Lys-Ac residue shows that gephyrin is acetylated *in vivo*. (**d**) Acetylation assay using FLAG–gephyrin, K666A, K715A, K219R or K666A+K715A site mutants in HEK293 cells. Denaturing IP for FLAG–gephyrin followed by WB against Pan Lys-Ac residue shows loss of acetylation in K666A and K666A+K715A mutants. Gephyrin levels after IP was determined by stripping the blot and WB using 3B11 antibody (lower panel). (**e**–**g'**) Morphology of eGFP–K666A and eGFP–K666Q mutants in primary hippocampal neurons compared with WT gephyrin. (**h**) Synaptic cluster density show similar distribution for eGFP–K666A and eGFP–K666Q compared with eGFP–gephyrin. (**i**) Mean size of eGFP–gephyrin and eGFP–K666A mutant is similar, but eGFP–K666Q has a significantly higher cluster size. Scale bar, 5 μm. Images from atleast four independent experimental replicates were analysed; error bars are s.e.m.

The *in vitro* SUMO assay using STREP-G and E domain peptides had suggested the presence of additional SUMO1 sites on E-domain. *In silico* analysis of gephyrin lists K373, K465, K602, K666 and K715 as potential SUMOylation sites (Supplementary Table 1). Although K373 and K465 residues are not surface-exposed, we did not find K602 to be an authentic SUMO site (Supplementary Fig. 1f). On the other hand, residues K666 and K715 are surface-exposed and lie close to the E-domain dimer interface. Hence, we tested whether K666 and K715 are SUMOylated. For this, we expressed and purified STREP-E-domain (wt) and STREP-E containing the K666A, K715A or K666A/K715A mutations from bacteria to perform *in vitro* SUMO1 and SUMO2 reactions as described above (Fig. 1a). STREP-E (wt) showed both SUMO1 and SUMO2 conjugation (Fig. 5a); however, STREP-E (K666A), STREP-E (K715A) or STREP-E (K666A/K715A) mutants did not exhibit any reduction in SUMO-1 or SUMO-2 conjugation (Fig. 5b), indicating that SUMOylation is unlikely to occur at these two residues.

Gephyrin can be acetylated at Lys residues (Lys-Ac)^16^. Hence, we probed whether either of these two residues that are strategically located on the E-domain are Lys-Ac. We confirmed gephyrin Lys-Ac *in vivo* by gephyrin IP from extracts of either the cerebral cortex or the cerebellum of adult mice and detection of Lys-Ac in WB using pan Lys-Ac antibodies and IgG as control. Gephyrin Lys-Ac band(s) were readily evident only in the gephyrin lane (Fig. 5c), confirming that gephyrin is acetylated *in vivo*. We next tested whether K666 and/or K715 might be relevant Lys-Ac sites. To determine the specificity of our assay, we mutated a nonspecific Lys residue in the unstructured linker domain (K219R), as SUMOylation and acetylation are restricted to structured domains. Whole-cell extracts of HEK293 cells transfected with FLAG–gephyrin, FLAG–K666A, FLAG–K715A, FLAG–K219R or FLAG–K666A/K715A were used for gephyrin IP using anti-FLAG antibody and probed for Lys-Ac in WB using pan Lys-Ac antibody (Fig. 5d). Lys-Ac bands were readily detected in lanes containing WT, K715R and K219R gephyrin mutants, but their intensity was reduced in both K666A and K666A+K715R gephyrin mutant lanes (Fig. 5d, lanes 3, 5 and 6 versus lanes 4 and 7), suggesting K666 is a major acetylation site on gephyrin. To ensure equal IP of gephyrin in all the lanes, we stripped the blot and re-probed using anti-gephyrin antibody 3B11 (Fig. 5d, lower lanes).

**Figure 6:**
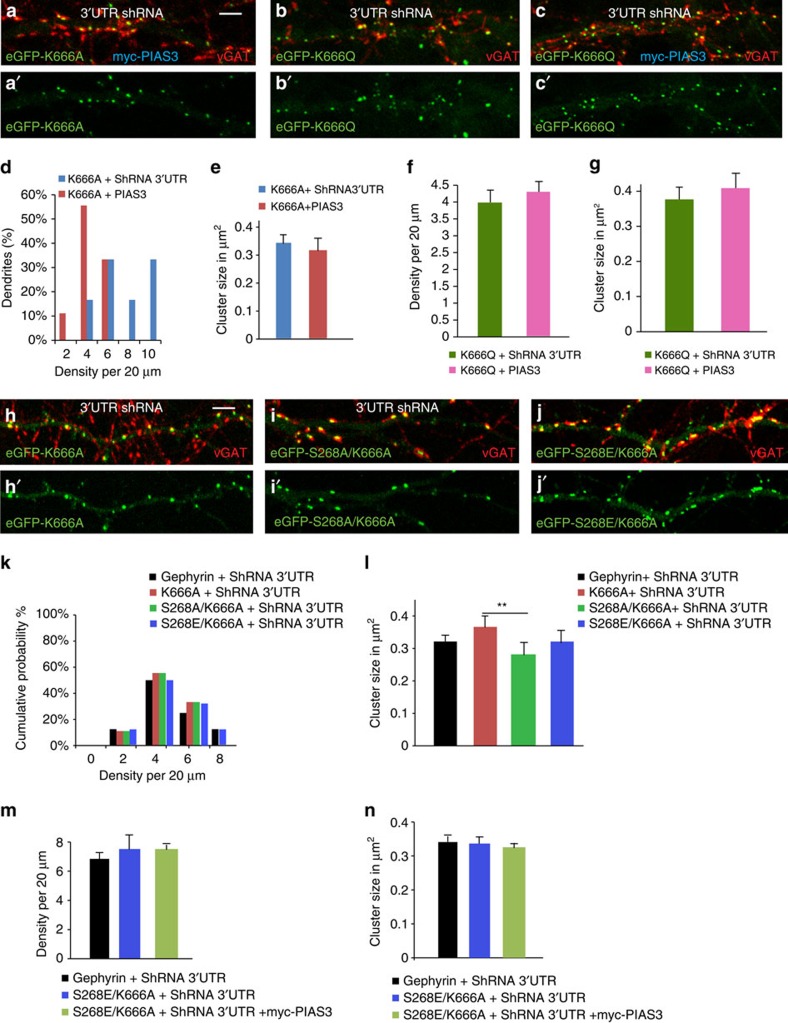
Cellular signalling pathways crosstalk. (**a**–**c'**) Morphology of neurons transfected with eGFP–K666A, eGFP–K666Q and 3′-UTR shRNA with or without myc–PIAS3. (**d**) Quantitative analysis shows cluster density distribution in neurons co-expressing eGFP–K666A and myc–PIAS3 is significantly decreased. (**e**) Mean gephyrin cluster size in neurons co-expressing eGFP–K666A and myc–PIAS3 shows no change. (**f**,**g**) eGFP–K666Q cluster density and cluster size is not altered in the presence of myc–PIAS3. (**h**–**j'**) Morphology of eGFP–K666A, eGFP–S268A/K666A or eGFP–S268E/K666A transfected neurons (DIV 8+7). (**k**) Synaptic cluster distribution shows no change in cluster density in neurons transfected with eGFP–K666A, eGFP–S268A/K666A or eGFP–S268E/K666A mutants. (**l**) Mean cluster size are not different between eGFP–gephyrin and eGFP–K666A and eGFP–K666A/S268A; mean cluster size is reduced between eGFP–K666A and eGFP–S268A/K666A. (**m**,**n**) myc–PIAS3 co-expression does not alter eGFP–S268E/K666A mutant phenotype. Scale bar, 10 μm. Images from atleast five independent experimental replicates were analysed; error bars are s.e.m.

Next, we investigated the importance of K666 acetylation for gephyrin postsynaptic clustering. We transfected neurons with eGFP–K666Q (Ac mimicking) or eGFP–K666A (Ac lacking) mutants along with 3′-UTR gephyrin shRNA and performed cluster analysis at DIV 8+7 relative to eGFP–gephyrin control (Fig. 5e–g'). Quantification of cluster density (5.1±0.57 versus 4.1±0.41 and 4.3±0.47, one-way ANOVA *F*_(2, 27)_=2.3, *P*=0.1) showed no significant differences. Comparison of cluster size (0.28±0.01 versus 0.4±0.01 μm^2^ and 0.34±0.01 μm^2^; one-way ANOVA *F*_(2, 604)_=11.4, *P*<0.0001; Bonferonni *post-hoc* test) revealed significant differences when K666 contains the acetylation mimicking mutation compared with WT control (Fig. 6g,h).

### Gephyrin acetylation influences PIAS3 effect

SUMOylation has been shown to be sometimes dependent on phosphorylation status of the substrate^8^. We wanted to test whether a link between acetylation and SUMOylation also exists. Hence, we co-transfected primary neurons with myc–PIAS3 and eGFP–K666A or eGFP–K666Q (acetylation mimicking), to evaluate whether the acetylation status at K666 might influence PIAS3 effect on gephyrin clustering (Fig. 6a–c'). Quantitative analysis revealed a significantly decreased eGFP–K666A cluster density (6.5±0.9 versus 3.8±0.4; Mann–Whitney test, *P*=0.003; Fig. 6d), but no change in cluster size (0.29 versus 0.24 μm^2^; Mann–Whitney test, *P*=0.6; Fig. 6e). On the other hand, acetylation mimicking K666Q mutation completely blocked all myc–PIAS3-induced changes in cluster size and density (Fig. 6f,g). This observation suggests that the acetylation status at K666 might influence PIAS3-dependent gephyrin SUMOylation.

Protein phosphorylation-dependent acetylation has been reported^21^. We thus determined whether acetylation of gephyrin at K666 might be coupled to phosphorylation at S268 site by generating eGFP–S268A/K666A and eGFP–S268E/K666A double mutants, and analysing their clustering in DIV 8+7 neurons (in the presence of gephyrin 3′-UTR shRNA; Fig. 6h–j'). Interestingly, eGFP–K666A/S268A combination mutation significantly reduced the cluster size compared with eGFP–K666A (0.32±0.01 versus 0.28±0.01; one-way ANOVA *F*_(4, 692)_=5.6, *P*=0.0037; Bonferonni *post-hoc* test; Fig. 6k); however, eGFP–S268E/K666A mutation had cluster size similar to eGFP–gephyrin control (Fig. 6j,j'). However, quantification for postsynaptic cluster densities of eGFP–gephyrin, eGFP–S268A/K666A, eGFP–S268E/K666A and eGFP–K666A did not exhibit significant differences (Fig. 6l; one-way ANOVA *F*_(4, 27)_=0.0024, *P*=0.9).

We wondered whether the PIAS3-induced change in cluster size and density (Fig. 3) could be blocked by eGFP–S268E/K666A mutation. We co-expressed myc–PIAS3, eGFP–S268E/K666A and gephyrin 3′-UTR shRNA in hippocampal neurons and found that eGFP–S268E/K666A mutation blocked all effect of PIAS3 on gephyrin cluster size and density change (Fig. 6m,n). These results reveal the importance of K666 and S268 sites for PIAS3 action on gephyrin. In conclusion, these experiments suggest that in addition to SUMO1 and SUMO2 modifications at K148 and K724, respectively, acetylation at K666 and phosphorylation at S268 are also important determinants of gephyrin-clustering properties.

Our data point towards a convergence of signalling cascades to regulate gephyrin scaffolding at GABAergic postsynaptic sites. However, it is unclear whether gephyrin cluster formation is a prerequisite for GABAergic synapse function. To test this principle, we generated eGFP–gephyrin dominant-negative (DN) mutant by introducing a premature stop codon after the K666 residue. This eGFP–gephyrin DN mutant exhibits a diffuse, non-clustered distribution in primary hippocampal neurons (Supplementary Fig. 4a). We examined the functional consequences of gephyrin scaffold disruption on GABAergic transmission, by recording GABAergic mIPSCs in neurons co-expressing eGFP–gephyrin DN and gephyrin 3′-UTR shRNA at either DIV 8+6 or DIV 11+5 (Supplementary Fig. 4b and Supplementary Table 3). Mock-transfected and eGFP–gephyrin control cells exhibited similar mIPSC amplitudes and inter-event intervals (Supplementary Fig. 4c,d). However, neurons expressing eGFP–gephyrin DN mutant showed significantly reduced mIPSC amplitudes and prolonged inter-event intervals (Supplementary Fig. 4c–f). Furthermore, the decay time constants of these events were also significantly longer compared with those measured in eGFP–gephyrin-transfected neurons (Supplementary Fig. 4g,h), suggesting that disruption of gephyrin scaffold might mainly affect either proximal synapses and/or specific subpopulation of GABA_A_Rs. Therefore, we morphologically tested for changes in the synaptic expression of GABA_A_Rs in eGFP–gephyrin or eGFP–gephyrin DN-expressing neurons. Although we could detect α2 subunit containing GABA_A_Rs in eGFP–gephyrin-transfected neurons, we saw a clear deficit in α2 and γ2 subunits in eGFP–K666A/S268E neurons (Supplementary Fig 4i–k, lower panels arrow heads). These results suggest that gephyrin scaffolding is essential for the surface expression of α2 subunit containing GABA_A_Rs.

### Cellular signalling pathways crosstalk

So far, our results show a morphological correlation between SUMO, acetylation or phosphorylation pathway(s). Hence, to obtain more direct evidence we performed proximity ligation assay (PLA) in primary hippocampal neurons transfected with eGFP–gephyrin. PLA allows for protein modification and/or interactions to be detected with high sensitivity and single-molecule resolution.

**Figure 7:**
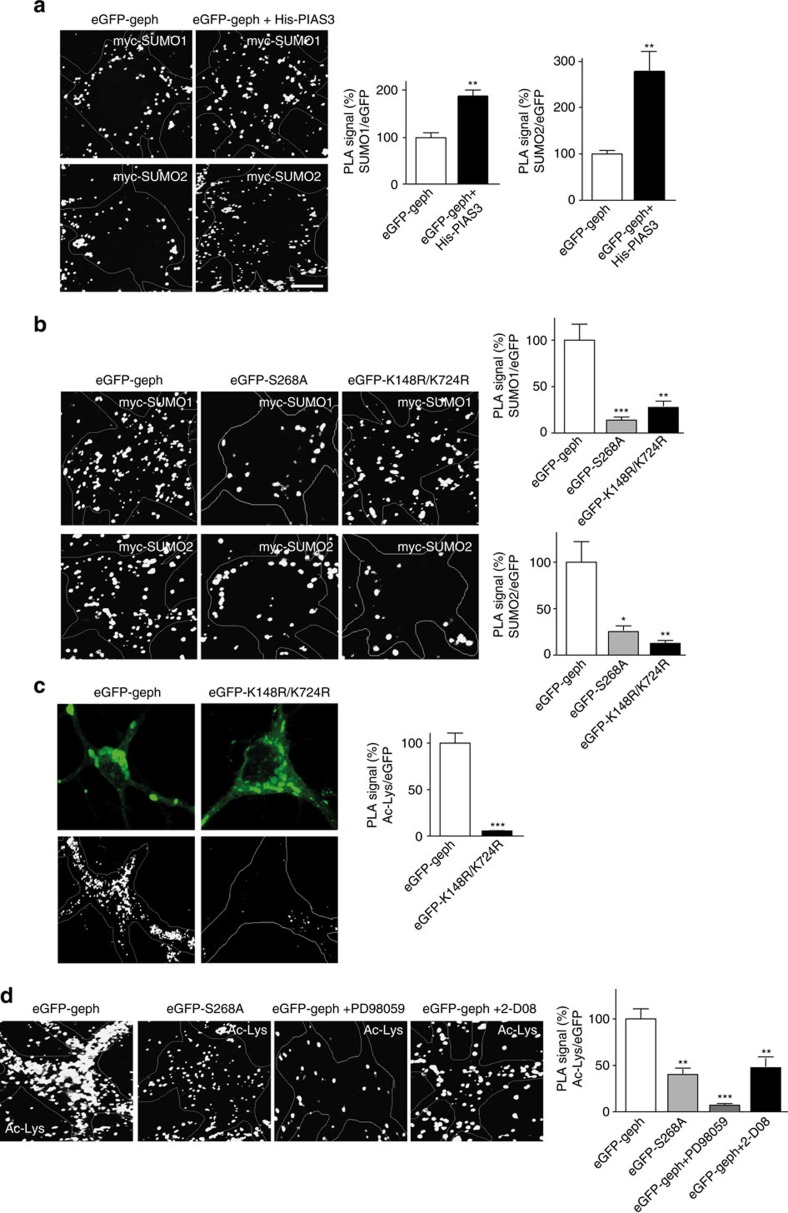
PLA assay to confirm convergence of cellular signalling pathways on gephyrin in neurons. (**a**) PLA signal for SUMO1 or SUMO2 conjugation on gephyrin in the presence or absence of His-PIAS3; (right panel) quantification of the PLA signal shows significant increase in SUMO1 and SUMO2 conjugation on gephyrin with co-expression of His-PIAS3. (**b**) PLA signal for SUMO1 or SUMO2 on eGFP–gephyrin, eGFP–S268A or eGFP–K148R/K724R mutants; (right panel) quantification shows significant reduction in SUMO1 and SUMO2 conjugation on eGFP–S268A or eGFP–K148R/K724R mutations. (**c**) eGFP signal and PLA signal for Ac-Lys on eGFP–K148R/K724R compared with eGFP–gephyrin; (right panel) quantification shows significant reduction of Ac-Lys conjugation on eGFP–K148R/K724R mutant. (**d**) PLA signal for Ac-Lys on eGFP–gephyrin, eGFP–S268A- and eGFP–gephyrin-transfected neurons treated with ERK1/2 inhibitor PD98059, and eGFP–gephyrin-transfected neurons with SUMO inhibitor 2-D08; (right panel) quantification of the Ac-Lys conjugation shows significant reduction when the ERK site is mutated or ERK pathway is pharmacologically blocked, or when SUMO conjugation on gephyrin is blocked. Images from three independent experimental replicates were analysed. Scale bar, 15 μm. Error bars are s.e.m.

Testing for direct SUMO1 or SUMO2 conjugation on eGFP–gephyrin confirmed our biochemical data. Furthermore, we could also confirm the direct influence of PIAS3 on gephyrin SUMO1 and SUMO2 conjugation using this assay (Fig. 7a). Consistent with our biochemical and morphological evidence, co-expression of His-PIAS3 showed a significant increase in SUMO1 and SUMO2 conjugation on eGFP–gephyrin (Fig. 7a, right panel; Student *t*-test, *P*=0.0008). Our data suggests a link between gephyrin phosphorylation at S268 residue and SUMOylation at K148 and K724 residues. To confirm the crosstalk between phosphorylation and SUMOylation pathways, we performed PLA using eGFP–gephyrin, eGFP–S268A or eGFP–K148R/K724R mutation and myc–SUMO1 or myc–SUMO2. Consistent with our other results, both eGFP–S268A and eGFP–148R/K724R mutations significantly reduce SUMO1 and SUMO2 conjugation on gephyrin (Fig. 7b; one-way ANOVA, *P*=0.0028).

Our results also predict a direct crosstalk between SUMO pathway and gephyrin acetylation. To confirm this we performed PLA using antibodies against enhanced green fluorescent protein (eGFP) and Ac-Lys residue. Although we could detect a strong Ac-Lys conjugation on WT eGFP–gephyrin control, we saw a significantly reduced Ac-Lys signal on our SUMO mutant eGFP–K148R/K724R (Fig. 7c, Student *t*-test, *P*<0.0001).

To confirm direct convergence of SUMO, ERK1/2 and acetylation pathways on gephyrin, we either used the eGFP–S268A mutation or pharmacological blockers for the ERK1/2 pathway (PD98059; 25 μM, 14 h) or SUMO pathway (2-D08; 50 μM, 14 h). Endogenous Ac-Lys conjugation on eGFP–S268A was significantly reduced. Furthermore, pharmacological blockade of ERK1/2 or SUMO pathways also reduced Ac-Lys conjugation on eGFP–gephyrin (Fig. 7d, one-way ANOVA, *P*=0.001). These results offer proof for the convergence of multiple signalling pathways on gephyrin scaffold, to regulate its clustering properties.

Next we used biochemistry to further confirm the link between phosphorylation, SUMOylation and acetylation pathways on gephyrin. For this we transfected eGFP–gephyrin, eGFP–K148R, eGFP–K724R, eGFP–K666A, eGFP–K666Q or eGFP–K148R/K724R into HEK293 cells and performed WB analysis using a gephyrin S270 phospho-specific antibody. Our analysis shows elevated levels of phospho-S270 in K724R (SUMO2) site mutation and significantly reduced phospho-S270 in K148R/K724R mutation (Supplementary Fig. 5). We also tested the interdependence of gephyrin phosphorylation on PIAS3 interaction. For this we co-transfected HEK293 cells with myc–PIAS3 and FLAG–gephyrin, FLAG–S268A, FLAG–S268E, FLAG–S270A or FLAG–S270E, followed by IP for myc–PIAS3 and WB against FLAG (Supplementary Fig. 5). Consistent with our PLA results we found a weaker interaction between PIAS3 and S268A mutation compared with WT; on the other hand, S270 site mutants bound better to myc–PIAS3 compared with S268 mutants or WT gephyrin. These biochemical observation is consistent with our morphology data showing eGFP–S270E but not eGFP–S268E mutant cluster size is influenced by PIAS3 (Fig. 4b,e).

### Pharmacological intervention and transgenic rescue *in vivo*

**Figure 8:**
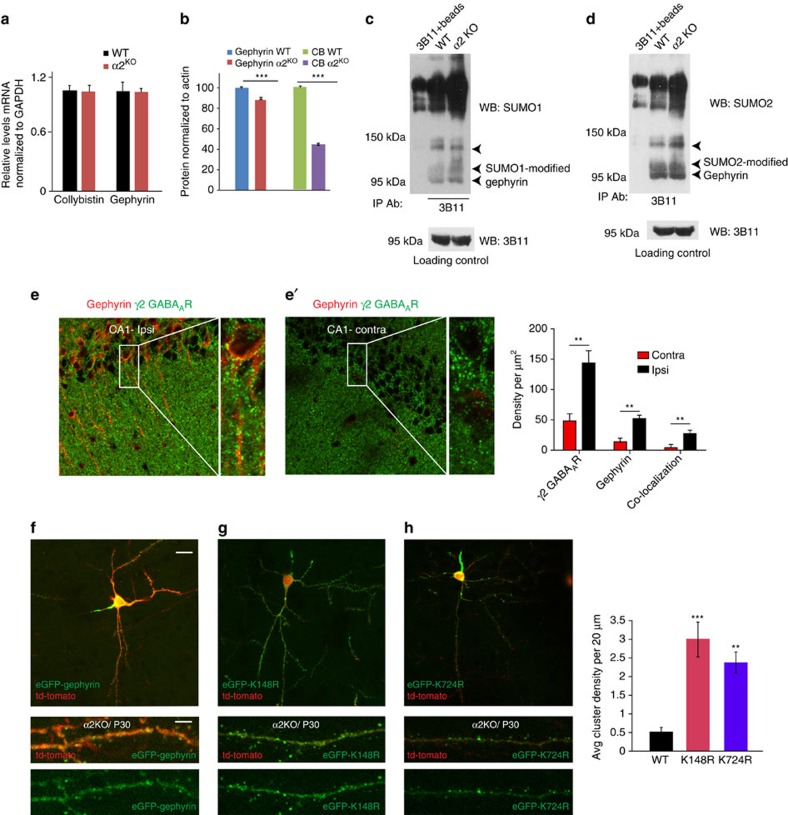
Gephyrin clustering in α2^−/−^ mice is rescued by blocking gephyrin SUMOylation. (**a**) Quantitative real-time PCR analysis of gephyrin and CB mRNA levels in WT and α2^−/−^ mouse brain (*n*=3 mice). (**b**) Quantification of WB assessing gephyrin and CB expression in brain homogenates from WT and α2 ^−/−^ mice (*n*=3 mice). (**c**,**d**) IP for gephyrin using whole-brain lysate from WT or α2^−/−^ mice and WB against SUMO1 or SUMO2 show higher level of gephyrin SUMO conjugation. The loading control for gephyrin expression levels in the IP samples is shown in the bottom panel. (**e**,**e'**) *In vivo* injection of SUMO inhibitor 2-D08 into α2^−/−^ and morphology analysis for gephyrin cluster colocalization with γ2 GABA_A_Rs; (right panel) quantification shows significant increase in gephyrin clustering along with γ2 GABA_A_R on the ipsi- compared with the contra-lateral hemisphere. (**f–h**) *In utero* electroporation of pCAG-LoxP-stop-LoxP-eGFP-gephyrin, pCAG-LoxP-stop-LoxP-eGFP-K148R or pCAG-LoxP-stop-LoxP-eGFP-K724R along with td-Tomato and ERT-Cre-ERT into E14 embryos and analysis of gephyrin clustering after induction with 4-OHT at P20 and visualization at P30 (*n*=5 mice) One-way ANOVA *P*<0.0001. Scale bars, 10 μm and 5 μm. Error bars are s.e.m.

Results so far add to the evidence that gephyrin is heavily, posttranslationally modified^16^ and the functional relevance for multiple PTMs on gephyrin remains unclear. If SUMOylation acts upstream of the phosphorylation and acetylation pathways, then gephyrin SUMO conjugation can be the rate-limiting step for scaffolding at GABAergic synapses. Hence, blocking the SUMO pathway in neurons where gephyrin scaffolding is disrupted should rescue scaffolding at GABAergic synapses. We have reported earlier that GABA_A_R α2 subunit-deficient mice exhibit a profound loss of gephyrin clustering in CA1 pyramidal cells^26^. The lead cause for this observed gephyrin scaffold loss is not known; therefore, we tested for transcriptional defects in gephyrin and CB, a RhoGEF important for gephyrin clustering and GABAergic synapse function^27^,^28^ in *Gabra2* knockout (KO) mice. We performed quantitative real-time PCR analysis from WT and α2^−/−^ mice brain tissue, and did not observe any significant differences in the transcript levels of gephyrin and CB (Fig. 8a), suggesting that loss of gephyrin clustering in α2^−/−^ mice is not due to reduced gephyrin transcription. Next, we assessed for gephyrin and CB protein levels using brain homogenates from WT and α2^−/−^ mice. We found 10% reduction in gephyrin protein in the absence of *Gabra2* gene; interestingly, we also found a 60% reduction in CB protein in α2^−/−^ mice (Fig. 8b).

The 10% loss of gephyrin protein in α2^−/−^ could not be a significant cause for the observed scaffolding loss. Hence, we wondered whether gephyrin PTM was adversely affected in the absence of functional α2 GABA_A_Rs at synaptic sites. We examined for difference in gephyrin SUMOylation between WT and α2^−/−^ mice. IP for gephyrin and WB for SUMO1 or SUMO2 consistently revealed stronger gephyrin SUMO1- and SUMO2-conjugated bands in *Gabra2* KO samples compared with WT control (Fig. 8c,d).

If enhanced gephyrin SUMOylation is indeed the main factor contributing to the scaffolding loss at GABAergic terminals, then pharmacological blockade of the SUMO pathway in α2^−/−^ cells should restore gephyrin scaffolding. We stereotactically injected SUMO pathway inhibitor 2-D08 (30 μM) or saline into α2^−/−^ mice (*n*=3) on one hemisphere near the hippocampal area. Twenty-four after injection, we analysed for gephyrin and γ2 GABA_A_R clusters in both ipsi- and contralateral hemispheres. One could see inflammation using antibody against CD68, a marker for microglia, near the lesion caused by the 2-D08 injection, but not saline. We imaged away from the lesion area to avoid tissue auto-fluorescence and nonspecific antibody reactivity. Quantification for gephyrin and γ2 GABA_A_Rs co-clustering showed a significant increase in density at the ipsi-lateral hemisphere in comparison with the contra-lateral control area (Student *t*-test, *P*=0.0025; Fig. 8e,e', right panel). The rescue of gephyrin scaffolding by blocking the SUMO pathway offers evidence for gephyrin SUMOylation to act upstream of the phosphorylation and acetylation pathways.

To obtain a more direct evidence for gephyrin scaffold recruitment downstream of α2 GABA_A_Rs via the modulation of SUMO pathway, we *in utero* co-electroporated LoxP-stop-LoxP-eGFP-gephyrin, LoxP-stop-LoxP-eGFP-K148R or LoxP-stop-LoxP-eGFP-K724R, along with tdTomato and ERT-Cre-ERT into E14 embryos of α2^−/−^ mice. We induced the expression of the gephyrin constructs at P20 using tamoxifen (4-OHT) and performed histological analysis at P30. Focusing on transfected neurons in layers 2 and 3 of parietal cortex, we observed in α2^−/−^ mice that neurons transfected with eGFP–gephyrin had a diffuse eGFP staining with only 2 to 3 clusters (Fig. 8f). However, α2^−/−^ neurons transfected with either of the SUMO mutants eGFP–K148R or eGFP–K724R showed distinct gephyrin clustering (Fig. 8g,h). The successful rescue of gephyrin clustering *in vivo* using either single point mutation of SUMO1 or SUMO2 site demonstrates that SUMO pathway converges onto gephyrin upstream of the phosphorylation and acetylation pathways. Furthermore, it also shows the specificity of our identified K148 and K724 residues for gephyrin scaffold regulation in neurons.

## Discussion

Scaffolding molecules in the postsynaptic density of glutamatergic synapses have canonical PDZ domains mediating multiple protein–protein interactions. However, gephyrin and its main interaction partners lack such an interaction domain, indicating that protein scaffolds at inhibitory synapses rely on distinct regulation mechanisms. A current view posits that a multi-molecular complex at inhibitory synapses is assembled via gephyrin self-aggregation. However, such a model fails to explain why gephyrin oligomerization does not occur nonspecifically and provides little insights into its regulation.

**Figure 9:**
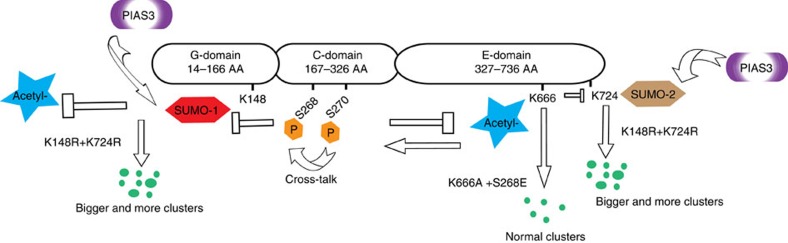
Model for gephyrin scaffolding at GABAergic synapses. Crosstalk between acetylation, phosphorylation and SUMOylation pathways regulate gephyrin scaffold formation at GABAergic postsynaptic sites.

In this study, we demonstrate how crosstalk between three different PTMs, SUMOylation, acetylation and phosphorylation mediates gephyrin clustering specifically at GABAergic postsynaptic sites (see model, Fig. 9). We posit that SUMOylation acts upstream of acetylation and phosphorylation. The formation of postsynaptic clusters is facilitated by de-conjugation of SUMO1 at K148 and SUMO2 at K724. Successful de-SUMOylation (presumably by members of the SENP protein family) leads to deacetylation at K666 and dephosphorylation at S268 residues to promote clustering. Furthermore, by identifying specific residues in gephyrin that influence its cluster size and density, we also posit that phosphorylation at S268 and deacetylation at K666 renders SUMOylation sites K148 and K724 ineffective in influencing gephyrin oligomerization. Quantitative analysis of gephyrin molecules using super-resolution microscopy showed that inhibitory postsynapse (PSD) comprises of 3,000–10,000 gephyrin molecules^29^. This study also identified micro-aggregates of gephyrin that are rapidly recruited to inhibitory postsynaptic sites for scaffold formation. Our study offers a model (Fig. 9) illustrating how SUMOylation, acetylation and phosphorylation act to recruit gephyrin molecules, to promote scaffolding based on the synapse requirement. By drawing on the importance of gephyrin phosphorylation at Ser270 and Ser268 residues to regulate cluster density and size^17^,^18^, the current study takes this understanding further by showing how PIAS3-mediated gephyrin SUMOylation could be dependent on S268 phosphorylation and K666 acetylation status (Supplementary Fig. 5). The importance of gephyrin acetylation has not been understood so far; our data demonstrates the central role of K666 acetylation for gephyrin SUMO conjugation and micro-cluster maintenance.

Although multiple GABA_A_R subunits have been shown to interact with gephyrin, it has become increasingly clear that gephyrin-independent GABA_A_R localization and clustering at synapses can occur. On the other hand, gephyrin itself depends on synaptic GABA_A_Rs to form postsynaptic clusters at inhibitory synapses in a neuronal cell-specific mechanism^16^. In *Gabra2*^−/−^ mice specific loss of gephyrin from CA1 pyramidal cells, but normal expression and function of α1 GABA_A_R, has been reported, suggesting that the α2 subunit has a fundamental influence on recruiting gephyrin at postsynaptic sites^26^.

In the current study, we rescue gephyrin clustering in *Gabra2*^−/−^ mice using both pharmacological blocker of SUMO and SUMOylation-defective gephyrin mutants, to demonstrate that modulation of this specific gephyrin PTM facilitates scaffolding at GABAergic postsynaptic sites. Further studies are required to uncover the molecular link between α2 GABA_A_Rs and gephyrin de-SUMOylation. It has been reported that *neuroligin-2* KO also leads to gephyrin cluster and GABAergic synapse loss from perisomatic region of neurons^14^. It is still unclear what proportion of neuroligin-2-containing synapses also contain α2 GABA_A_Rs, especially given that in *Gabra2* KO mice we also see gephyrin cluster loss in the stratum radiatum of CA1. The significance of gephyrin PTMs for regulating GABAergic transmission is supported by another independent study showing gephyrin dephosphorylation at S270 residue regulates dendrite growth and branching by altering GABAergic, but not glutamatergic transmission^30^.

Our current study identifies SUMO1 and SUMO2 co-localized with gephyrin clusters at GABAergic synapses. We observed three distinct subcellular distributions of SUMO1/2 proteins in neurons. When SUMO proteins were enriched in dendrites, we observed co-localization with eGFP–gephyrin at VGAT-positive terminals (Supplementary Fig 3a–b''). It is possible that specific cellular signal(s) regulate SUMO subcellular localization within neurons, thus contributing to differences in synaptic responses within a given network. However, the exact nature of such a signalling factor is currently unclear.

In the current study we also identified additional SUMO1 conjugation sites on gephyrin E domain. It is likely to be that more than one site is SUMO2 conjugated on gephyrin, opening up possibilities for diverse regulatory effects of SUMO on gephyrin subcellular localization and function. In addition, our data highlight a currently unknown role for GABA_A_Rs in influencing gephyrin SUMO conjugation for scaffold recruitment and synapse function.

Identification of diverse gephyrin-interacting molecules and their role in gephyrin clustering led to at least two different models, both implicating CB and emphasizing the need of specific GABA_A_Rs subunits for scaffold formation^16^. In addition, GABA_A_R PTM is known to play an important role in regulating GABAergic synapse function and plasticity. Phosphorylation of specific receptor subunits has been shown to influence surface trafficking, lateral diffusion to and from synaptic sites along the plasma membrane, and receptor internalization^31^,^32^,^33^. It is rather unlikely, in our view, that changes in gephyrin-scaffolding properties and receptor trafficking/function via PTMs are mutually exclusive. Rather, we propose that specific currently unknown neurotrophic factor(s) signal downstream to recruit specific kinases, phosphatases, acetyl-transferase and so on to inhibitory postsynaptic sites, to influence GABAergic synaptic plasticity by acting in concert on the gephyrin scaffold and the receptors anchored to it.

Finally, gephyrin PTM-dependent scaffolding dynamics could act as a signalling hub integrating heterosynaptic activity, to adjust the strength of GABAergic transmission. We have recently reported that NMDA (*N*-methyl-D-aspartate) receptor-dependent neuronal depolarization regulates gephyrin phosphorylation in a CaMKII-dependent mechanism, to upregulate perisomatic inhibition and facilitate neuronal homeostasis^34^. Experimentally, mutations in GABA_A_R subunit genes that indirectly affect postsynaptic gephyrin clustering have been used to generate models for neurodevelopmental and neuropsychiatric disorders^35^,^36^. In light of our data demonstrating the importance of gephyrin scaffolding, these perturbations at the receptor levels could induce alterations in neuronal network by the disruption of gephyrin signalling hubs at inhibitory postsynaptic sites.

## Methods

### Bacterial expression vectors

IBA7-STREP-gephyrinP1, IBA7-STREP-E-domain and IBA7-STREP-G-domain have been described before^17^. pGEX-E1, pET28a-His-Ubc9, pET28a-His-SUMO1GG and pET28aHis-SUMO2GG are described earlier^37^. IBA7-STREP (K666A, K715A) and IBA7-STREP-K715A+K666A were created in a eGFP–gephyrin P1 template using site-directed mutagenesis PCR, following the manufacturer's instructions (Stratagene) and the mutations were sequence confirmed. Gephyrin sequence was subcloned into IBA7 vector using EcoRI and KpnI sites. IBA7-STREP-G peptides (1–82, 83–166 and 40–120) and IBA7-STREP-E peptides (326–454, 455–558, 559–634, 635–736) were generated by PCR amplification using eGFP–gephyrinP1 as template and subcloned into IBA7 vector using EcoRI and KpnI sites. Numbering refer to the amino acid number in the rat gephyrin-P1 complementary DNA (NCBI Reference Sequence: NP 074056.2). IBA7-STREP-G1-82 (K67N, K57R), IBA7-STREP-G40-120 (K77R, K101R), IBA7-STREP-G83-166 (K148R) and IBA7-STREP-G K148R mutants were created by site-directed mutagenesis PCR using respective WT template and the mutations were sequence confirmed. Similarly, IBA7-E326-454, IBA7-E455-558, IBA7-E559-634 and IBA7-E635-736 were also generated by PCR amplification using eGFPC2–gephyrin as template and subcloning the PCR fragment into IBA7 vector using EcoRI and KpnI sites. IBA7-E326-454 (K328R, K362R and K373R), IBA7-E455-558 (K497R, K521R and K473R) and IBA7-E635-736 (K724R and K645R) were generated using site-directed mutagenesis and sequence confirmed.

### Neuronal expression vectors

eGFP–Gephyrin P1 has been described before^17^. pCR-HA-SUMO1 and pCR-HA-SUMO2 are described in ref. 38; pCDNA3–myc SUMO1/2, pCI-His-PIAS3 and pCMV-6xmyc-PIAS2β have been described earlier^37^. pmU6Pro-gephyrin shRNA (3′-UTR and 3′-UTR-3m) have been described earlier^17^,^24^. eGFP (gephyrin, S268A, S268E, S270A and S270E) have been described earlier^17^,^18^. eGFP–GephyrinP1 K148R, K724R, K148R/K724R, K666A, K666Q, S268A/K666A, S268E/K666A were obtained from eGFP–GephyrinP1 template by site-directed mutagenesis PCR and sequence confirmed. pCAG-ERT2Cre tamoxifen-inducible Cre recombinase has been described earlier^39^. pCAG-LoxP-Stop-LoxP-GFP-Gephyrin has been described earlier^39^. pCAG-LoxP-Stop-LoxP-GFP-K148R and pCAG-LoxP-Stop-LoxP-GFP-K724R were created by subcloning into pCAG-LoxP-Stop-LoxP-GFP-Gephyrin plasmid after removing GFP–gephyrin using NheI and KpnI, and sequence confirmed.

### Antibodies

The antbodies used are as follows: rabbit anti-STREP horseradish peroxidase (1:10,000, BioRad, 161-0380), Mouse anti-Gephyrin 3B11 (1:1,000, SySy, 147111), mouse anti-gephyrin mAb7a (1:2,000, SySy, 147021), rabbit anti-vGAT (1:3,000, SySy, 131003), mouse anti-vGAT (1:5,000, SySy, 131011), rat anti-HA (1:3,000, Roche), mouse anti-Myc (1:5,000, Roche, 11667149001), mouse anti-FLAG (1:5,000, Sigma-Aldrich, F3165), rabbit anti-GFP (1:5,000, Synaptic Systems, 132002), chicken anti-GFP (1:5,000, Chemicon, AB16901), mouse anti SUMO-1 (1:1,000, 21C7, Hybridoma bank), mouse anti SUMO2/3 (1:1,000, 8A2, Hybridoma Bank), rabbit anti-SUMO1 (1:250, Epitomics, 1563-1), rabbit anti-SUMO2/3 (1,250, Epitomics, 2970-1), rabbit anti pan-Acetylated Lysine antibody (1:1,000, Cell Signaling, 9441) and mouse anti-Ac-Lysine (AKL5C1) (1:2,000, Santa Cruz, Sc32268). Gephyrin anti-S270 phospho-antibody has been described earlier^28^. The following secondary antibodies were used: goat anti-mouse IgG Cy3 (1:500, Jackson Immunoresearch, 115165), goat anti-mouse IgG Cy5 (1:500, Jackson Immunoresearch, 115175), goat anti-rabbit IgG Cy3 (1:500, Jackson Immunoresearch, 111165), goat anti-rabbit IgG Cy5 (1:500, Jackson Immunoresearch, 111175), goat anti-mouse IgG alkaline phosphatase (AP) (1:30,000, Sigma-Aldrich, A3562) and goat anti-rabbit IgG AP (1:30,000, Sigma-Aldrich, A3687).

### Cell lines and transfection

HEK293T cells (ATCC, CRL 11268) tested negative for mycoplasma and were cultured at 37 °C, 5% CO_2_ in DMEM medium supplemented with 5% fetal bovine serum and were transfected with 3–6 μg DNA 14 h postplating using polyethylenimine (Polyscienes, Inc.) according to the manufacturer's recommendation. The cells were lysed 12–24 h post transfection for analysis.

### IP and WB analysis

Brain homogenates were prepared as described earlier^17^. IP analysis was performed using (1–2 μg) antibodies and the protein complexes were collected using 30 μl protein G agarose beads. The bound complexes were washed in high-salt buffer and twice in EBC buffer (50 mM Tris pH 8.0, sodium chloride 120 mM and NP-40 0.5%). Washed beads were boiled 5 min, 90C in 2 × Simple Buffer (SDS and 15% β-mercaptoethanol) and using SDS–PAGE at 140 V, room temperature (RT). Proteins were transferred on a polyvinylidene fluoride membrane (37 V, 90 min), blocked in 5% western blocking reagent (Roche Diagnostic) for 10 min and incubated 1 h with primary antibody solution. Membrane was washed three times in 1 × TBST (50 mM Tris-HCl, 150 mM NaCl and 0.05% Tween-20) and incubated with secondary antibody linked to AP or horseradish peroxidase, to allow visualization of the protein bands.

### Bacterial overexpression of proteins and purification

Bacteria (BL21; pLyss) expressing the appropriate plasmid of interest were grown in 500 ml of media to OD_600_=0.6. Protein expression was induced using 1 mM anhydrotetracycline or 1 mM isopropyl-β-D-thiogalactoside for 4 h, 30 °C and pelleted (11,000 r.p.m., 20 min). Bacterial pellet was lysed in EBC lysis buffer containing cOmplete mini protease inhibitor cocktail (Roche Diagnostic) and 2 mg ml^−1^ lysozyme (AppliChem, A37110001). Bacterial cells were broken by sonication (ten cycles of 30 s each). The clear cell lysate was collected by centrifugation (18,000 r.p.m., 30 min, 4 °C) and proteins purified using FPLC (Amersham) system, immobilized metal ion affinity chromatography.

Bacteria expressing pGEX-SAE1/2 plasmid were induced with isopropyl-β-D-thiogalactoside and lysed using the lysis buffer: 50 mM NaH2PO4 pH 8, 300 mM NaCl, 10% glycerol, 0.5% Tween-20, 5 mM dithiothreitol (DTT), cOmplete mini protease inhibitor cocktail (Roche Diagnostic) and 2.5 mM imidazole. This contained a His-tag, a GST-tag and a thrombin cleavage site, and was purified using the NiNTA column and Glutathione Sepharose column using FPLC as described in the manufacturer's instructions (GE Healthcare). Elution was done using 10CV elution buffer: 50 mM NaH_2_PO_4_ pH 8, 300 mM NaCl, 10% glycerol, 0.5% Tween-20, 5 mM DTT and 250 mM imidazole, collected fractions were dialysed again with PBS pH 7.4, 0.1% Triton X-100 and 5% glycerol, and incubated on 2 ml GST-Sepharose (GE healthcare) for 1 h, 4 °C, rotor. Bead were washed twice in PBS and 25 cleavage unit of thrombin (GE Healthcare) were applied to a total volume of 1.5 ml PBS and incubated overnight at 37 °C on the rotor. Cleaved fraction was purified using 2 ml Ni-Sepharose bead volume, washed, eluted and dialysed using the same protocol as in the first run of purification. Aliquots were stored at −80 °C. Purified proteins were examined for purity and expression levels using Coommasie staining of SDS gel.

### *In vitro* SUMOylation assay

STREP-gephyrin, STREP-G, STREP-E or STREP-gephyrin peptides were immobilized using Strep-tactin sepharose beads (IBA GmbH, 40 μl per 100 μl lysate) for 30 min, batch purified using EBC buffer and equilibrated in SUMO buffer (50 mM Tris-HCl pH 8, 50 mM NaCl, 5 mM MgCl_2_, 10% glycerol and 0.5 mM DTT) before *in vitro* SUMOylation. Equal amounts of bacterial expressed and purified E1, Ubc9 and SUMO1 or SUMO2 were added to the tubes and reaction started using 0.3 μl of 5 mM ATP by incubating at 32 °C, 100 min. Supernatant was discarded and beads were washed once in EBC buffer before boiling the beads in 15 μl of 2 × Simple Buffer for 5 min at 90 °C.

### Primary neuronal cultures and transfection

All *in vivo* experiments were approved by the Cantonal Veterinary Office of Zurich. Primary hippocampal neuronal cultures from E17 rat embryos as described earlier^17^,^18^. The cells were transfected by Lipofectamine 2000 (Life Technology) and CombiMag (OZ Biosciences) as described earlier^40^, using 1 μg total plasmid DNA and incubated for 1 week (37 °C, 5% CO_2_). Seven days post transfection (DIV8+7), neurons were rinsed in PBS pH 7.4 and immediately fixed in 4% paraformaldehyde (PFA) for 15 min at RT and stained with appropriate antibodies.

### Imaging of primary neurons

All the image acquisition and analysis was done blind by different students to avoid experimenter bias. The images were analysed from at least three to five independent transfections. Immunofluorescence data were acquired using confocal laser scanning microscope (LSM 700, Carl Zeiss) using × 40 lens (numerical aperture 1.4) and pinhole setup to 1 Airy unit and pixel size of 90 nm. At least ten cells from three independent cultures were used for each treatment and imaged as a *z*-stack (three to four optical sections) and 0.5 μm step length. Image analysis and quantification were performed using ImageJ software (http://rsb.info.nih.gov/ij/), maximum intensity projections were created from the *z*-stacks for analysis. Clusters were defined using automatic threshold algorithm to select region of interest. The Analyze Particles function in ImageJ was used to count the number of cluster (area>0.04 μm^2^) and measure their size (area in μm^2^). To determine apposition to presynaptic terminal (vGAT puncta, secondary channel), the size of the cluster was increased by 1 pixel all around. Apposition between the threshold secondary channel and selected regions of interest in the primary channel was considered as synaptic gephyrin cluster if apposition was greater than 0.010 μm. Analysis of the generated data was plotted using Exel software (Microsoft). Statistical significance was analysed using Prism software.

### Electrophysiology

Whole-cell voltage-clamp recordings were performed in hippocampal cultured neurons (8+6 DIV) at RT and at a holding potential of −60 mV. Patch electrodes (3–4 MΩ) were pulled from borosilicate glass and filled with (in mM) 120 CsCl, 10 EGTA, 10 HEPES pH 7.4, 4 MgCl_2_, 0.5 GTP and 2 ATP. The external solution contained (in mM) 150 NaCl, 10 KCl, 2.0 CaCl_2_, 1.0 MgCl_2_, 10 HEPES pH 7.4 and 10 glucose. Recordings were performed using a HEKA EPC-7 amplifier and Patch Master v2.11 software (HEKA Elektronik, Germany). Spontaneous GABAergic miniature postsynaptic currents (mIPSCs) were isolated by adding CNQX (2 μM, Tocris), AP-5 (50 μM, Tocris) and tetrodotoxin (0.1 μM, Tocris). After 3 min of establishing the whole-cell mode, mIPSCs occurring during a 1 min interval and displaying amplitudes above the background noise (6–12 pA) were identified and analysed offline using MiniAnalysis 6.0.7 software (Synaptosoft). The decay phase of mIPSCs was fitted with a single exponential curve and both rise and decay phase were fitted between 10 and 90% of its amplitude. For the average amplitude calculation, only cells with more than 25–30 events in the recording period were included. However, for the calculation of inter-event interval, cells with lower number of events were included, as these may represent a loss of postsynaptic clusters.

### Proximity ligation assay

*In situ* PLA was performed using the DuoLink II kit (Olink Bioscience) according to the instructions of the manufacturer. The primary antibody solution contained rabbit or mouse anti-GFP and rabbit or mouse anti-Ac-Lys, anti-myc. The primary neurons in the coverslips were fixed using 4% PFA and later permeabilized using 0.2% Triton X-100. They were incubated in primary antibody solution overnight at 4 °C. The secondary antibodies conjugated to oligonucleotides, PLA probes anti-Mouse MINUS and anti-Rabbit PLUS (Duolink II, Olink Bioscinece) were diluted 1:5 in 10% normal goat serum (NGS) (40 μl of PLA probe solution are needed per coverslip) and incubated for 20 min at RT. Later, 40 μl of PLA probe solution was pipetted on top of each coverslip followed by incubation for 1 h at 37 °C. The ligation solution was prepared (Ligation (5 × ) stock solution, 1:5 in H_2_O, Duolink II (Olink Bioscience); Ligase (1 U μl^−1^) stock solution, 1:40 in H_2_O, Duolink II (Olink Bioscience)). The ligation solution was added to the coverslips (40 μl per coverslip) and incubated for 1 h at 37 °C. The coverslips were then washed two times for two minutes in Duolink II Wash Buffer A. The amplification solution (Amplification Red (5 × ) stock solution, 1:5 in H_2_O, Duolink II (Olink Bioscinece); Polymerase (10 U μl^−1^) stock solution, 1:80 in H_2_O, Duolink II (Olink Bioscience)) was prepared and added. The coverslips were washed two times for 10 min in Duolink II Wash Buffer B at RT. Finally, coverslips were mounted on microscope slides with fluorescence mounting medium (Dako), to reduce fading of immunofluorescence during microscopy.

### Stereotactic brain surgery and 2-D08 injection

All *in vivo* experiments were approved by the Cantonal Veterinary Office of Zurich.

Eight- to 10-week-old mice (both male and female) were used for the study. A 5 cm-long glass pipette (Ø *ca*. 50–60 μm at the tip) was buffered with Mineral Oil (Sigma Cell Culture, *d*=0.84 g ml^−1^) and then attached to the injection apparatus (Drummond Scientific Company) to take up 10 μl of 2-D08 (Sigma, 30 μM) or NaCl.

To anaesthetize the mice, they were put in a closed box (Indulab AG, Gams, CH) with a gas supply, where isoflurane diluted in O_2_ (mixer used, Northern vaporizers) was introduced. Once the animal did not respond to common pain stimuli anymore, the head was stably fixed in place in the Kopf stereotaxic setup (Model 900 Small Animal Stereotaxic Instrument). During surgery, the isoflurane concentration was reduced to 2–4%. Mice were kept on a warm pad during and after surgery, to prevent a drastic cooling of the body temperature. To prevent eyes from drying out, they were covered with Vitamin A (Bausch&Lomb Swiss AG).

Craniotomy was performed in the right hemisphere (stereotactic coordinates: anteroposterior −1.8 mm, lateral +1.6 mm, dorsoventral +1.9 mm) using a drill (Model SR, Foredom Electric Co., Bethel, CT, USA). Mice received a unilateral stereotaxic injection of 70 nl 2-D08 or NaCl into the dorsal hippocampus. To allow 2-D08 (or NaCl) diffusion, there was a 2 min interval between the injection and the removal of the capillary.

Afterwards, the skin was sewed with three stitches and a monofibrillar suture. Mice were intraperitoneally (i.p.) injected with 1 mg kg^−1^ buprenorphine Temgesic (Reckitt Benckiser, CH, LOT 5348, 1:10 in NaCl) and were then allowed to recover in a separate, warmed cage until they resumed their normal behaviour. The mice were perfused 24 h post injection post surgery for morphology analysis.

### *In-utero* electroporation and tissue preparation

All *in vivo* experiments were approved by the Cantonal Veterinary Office of Zurich.

Timed-pregnant mothers (14 days) were anaesthetized with isoflurane in oxygen carrier. Uteri were exposed through a 2 cm incision in the ventral peritoneum. Embryos were carefully lifted using ring forceps through the incision and placed on humidified gauze pads. pCAG-LoxP-Stop-LoxP-eGFPC2-gephyrin, pCAG-LoxP-Stop-LoxP-eGFPC2-K148R and pCAG-LoxP-Stop-LoxP-eGFPC2-K724R were co-injected with pCAG-LoxP-Stop-LoxP-tdTomato and pCAG-ERT2CreERT2 (total concentration 3 μg μl^−1^, prepared using Endo Free plasmid purification kit) mixed with 0.05% Fast Green (Sigma) was injected through the uterine wall into the telencephalic vesicle using pulled borosilicate needles and a Femtojet microinjector (VWR). Five electrical pulses were applied at 50 V (50 ms duration) across the uterine wall at 1 s intervals using 5 mm platinum tweezer electrodes (CUY650P5, Nepagene) and an ECM-830 BTX square wave electroporator (BTX, Gentronic, Inc.). The uterine horns were then replaced in the abdominal cavity, and the abdomen wall and skin were sutured using surgical needle and thread. The pregnant mouse was injected with buprenorphine (Vetergesic; Alstoe Ltd) and warmed on heating pad until it woke up. The whole procedure was complete within 30 min. Conditional expression of the electroporated proteins was archived by i.p. administration of 50 μl 1 mM tamoxifen at postnatal day 10. Mice were anaesthetized with pentobarbital (Nembutal; 60 mg kg^−1^, i.p.) and perfused transcardially with 20 ml PBS pH 7.4 to flush the blood away at P15, followed by 60 ml ice-cold fixative solution (4% PFA, 0.15 M NaH_2_PO_4_, 15% saturated picric acid pH 7.4). After perfusion, brains were rapidly dissected and postfixed in 50 ml using 4% PFA for 3 h and rinsed with PBS before transferring to 30% sucrose in PBS overnight 4 °C. The brains were frozen in powered dry ice and stored at −80 °C. The brains were cut with the sliding microtome 40 μm-thick and stored in anti-freeze solution (50 mM phosphate buffer, 15% glucose, 30% v/v ethylene glycol, sodium azide pH 7.4) and stored at −20 °C. Sections, stored in anti-freeze solution, were washed twice for 10 min in Tris-buffered saline (pH 7.4) in 12-well culture plates. The sections were incubated in primary antibody diluted in PBS pH 7.4 containing 2% NGS and 0.2% Triton X-100 using nine-well glass plates in a moist chamber overnight at 4 °C at 100 r.p.m. The day after, sections were washed three times 10 min in PBS pH 7.4 and incubated in secondary antibodies diluted in PBS pH 7.4 containing 2% NGS for 30 min at RT in shaker. The sections were washed again three times 10 min in PBS pH 7.4, mounted on gelatinized glass slides and mounted using DAKO mounting media for confocal image analysis.

### Data availability

All data generated or analysed during this study are included in this published article (and its Supplementary Information files) or are available from the author upon request.

## Additional information

**How to cite this article:** Ghosh, H. *et al*. Several posttranslational modifications act in concert to regulate gephyrin scaffolding and GABAergic transmission. *Nat. Commun.* **7,** 13365 doi: 10.1038/ncomms13365 (2016).

**Publisher's note:** Springer Nature remains neutral with regard to jurisdictional claims in published maps and institutional affiliations.

## Supplementary Material

Supplementary Information Supplementary Figures 1- 5 and Supplementary Tables 1 - 3
