# Supplementary material for: Several posttranslational modifications act in concert to regulate gephyrin scaffolding and GABAergic transmission
Source: Nat Commun. 2016 Nov 7;7:13365. doi: 10.1038/ncomms13365 (PMC5103071; doi:10.1038/ncomms13365)
Supplement: Supplementary Information — Supplementary Figures 1- 5 and Supplementary Tables 1 - 3 [file ncomms13365-s1.pdf]

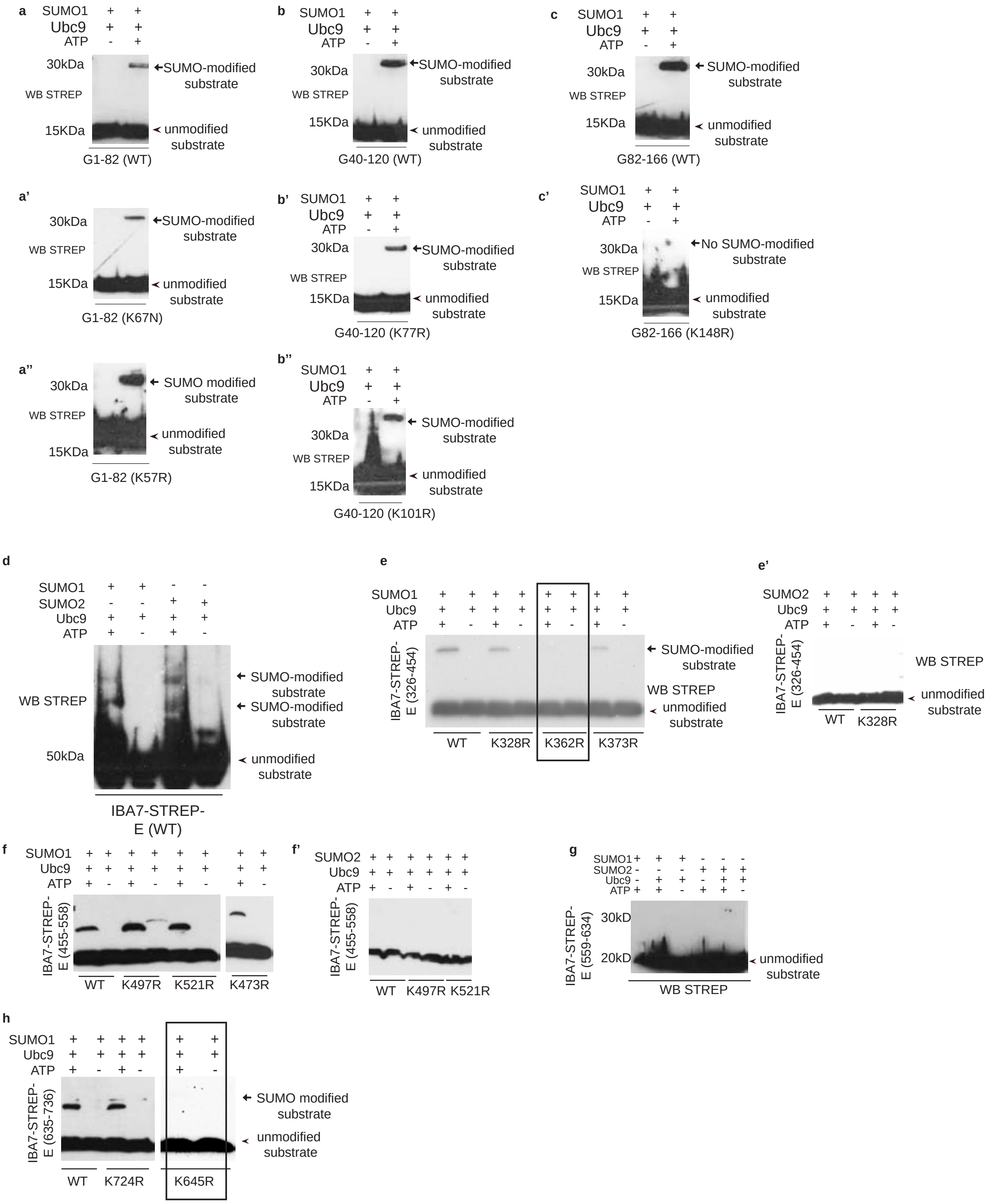

Suppl. Fig. 1: In vitro SUMO assay to identify SUMO-1 and SUMO-2 sites on gephyrin G and E domains. (a) STREP-G (1-82) peptide shows SUMO-1 conjugation. (a') STREP-G (1-82) K67N does not abrogate SUMO-1 conjugation. (a") STREP-G (1-82) K57R shows SUMO-1 conjugation. (b) STREP-G (40-120) showing SUMO-1 conjugation. (b') STREP-G (40-120) K77R mutant also shows SUMO-1 conjugation suggesting this is not the SUMO-1 site. (b") STREP-G (40-120) K101R mutant shows SUMO-1 conjugation suggesting this is not a SUMO-1 site. (c) STREP-G (82-166) shows SUMO-1 conjugation. (c') STREP-G (82-166) K148R mutant abrogates SUMO-1 conjugation suggesting this is a SUMO-1 site. (d) STREP-E domain shows both SUMO1 and SUMO2 conjugated bands. (e) STREP-E (326-454), STREP-E (326-454) K328R, K373R mutant showing a SUMO-1 conjugation, but K362R mutant does not show SUMO1 conjugation. (e') STREP-E (326-454) and STREP-E (326-454) K328R do not conjugate SUMO-2. (f) STREP-E (455-558), STREP-E (455-558) K497R, K512R, K473R mutants show SUMO-1 conjugation. (f') STREP-E (455-558), STREP-E (455-558) K497R, K512R mutants do not show SUMO-2 conjugation. (g) STREP-E (559-634) does not get conjugated with SUMO-1 or SUMO-2. (h) STREP-E (635-736), STREP-E (635-736) K724R show SUMO1 conjugation, but K645R does not show SUMO1 conjugation.

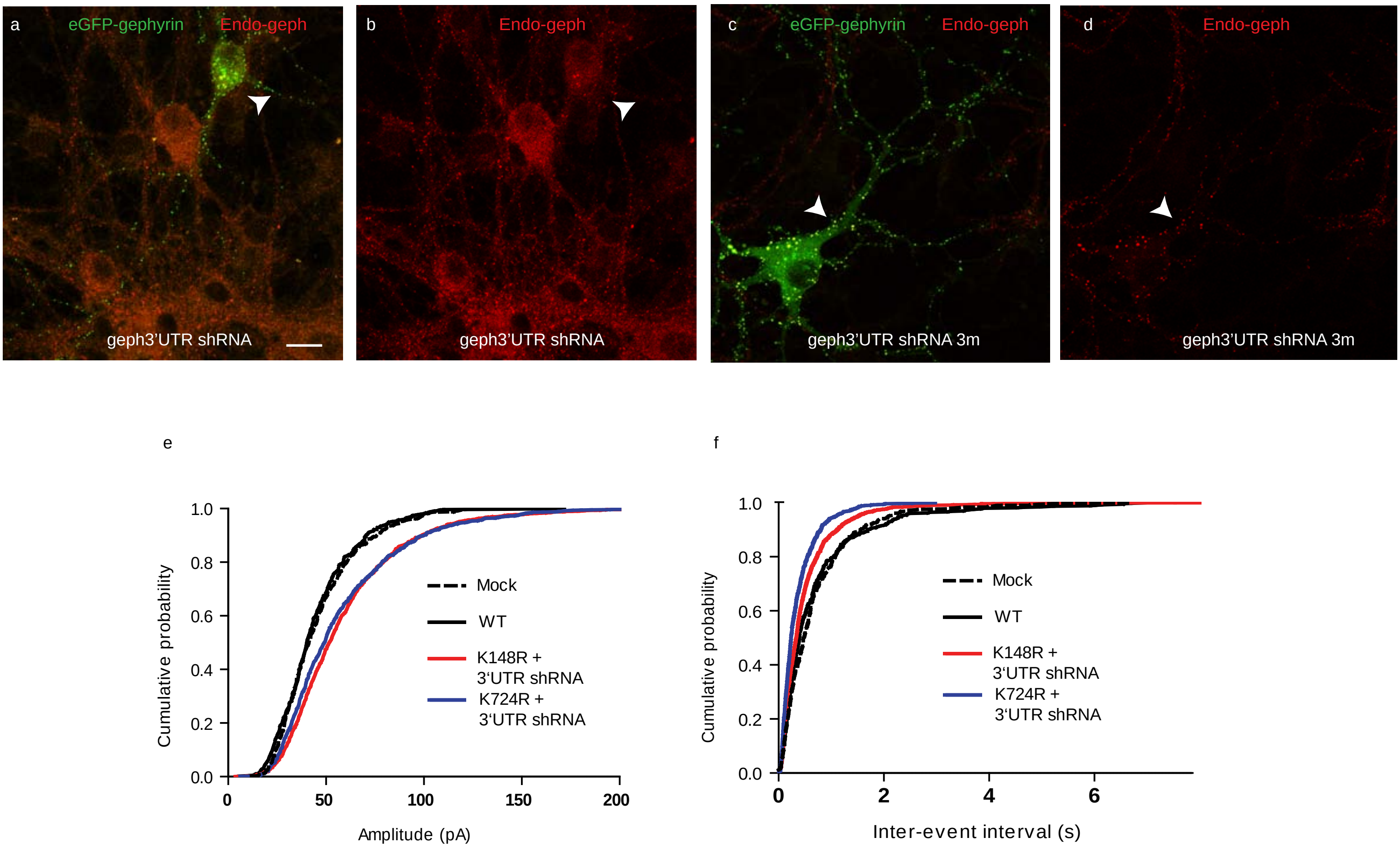

Suppl. Fig. 2: Functional analysis of neurons transfected with gephyrin SUMO mutants. (a-d) Down regulation of endogenous gephyrin clusters using shRNA against 3'UTR sequence. Arrow head shows loss of endogenous gephyrin puncta in neurons transfected with shRNA but not shRNA 3m variant sequence. (e) Cumulative probability distribution of mIPSC amplitude of mock transfected neurons or neurons co-transfected with WT, eGFP-K148R or eGFP-K724R along with gephyrin 3'UTR shRNA. (f) Cumulative probability distribution of mIPSC inter-event interval is significantly reduced in neurons expressing eGFP-K148R or eGFP-K724R SUMO mutants.

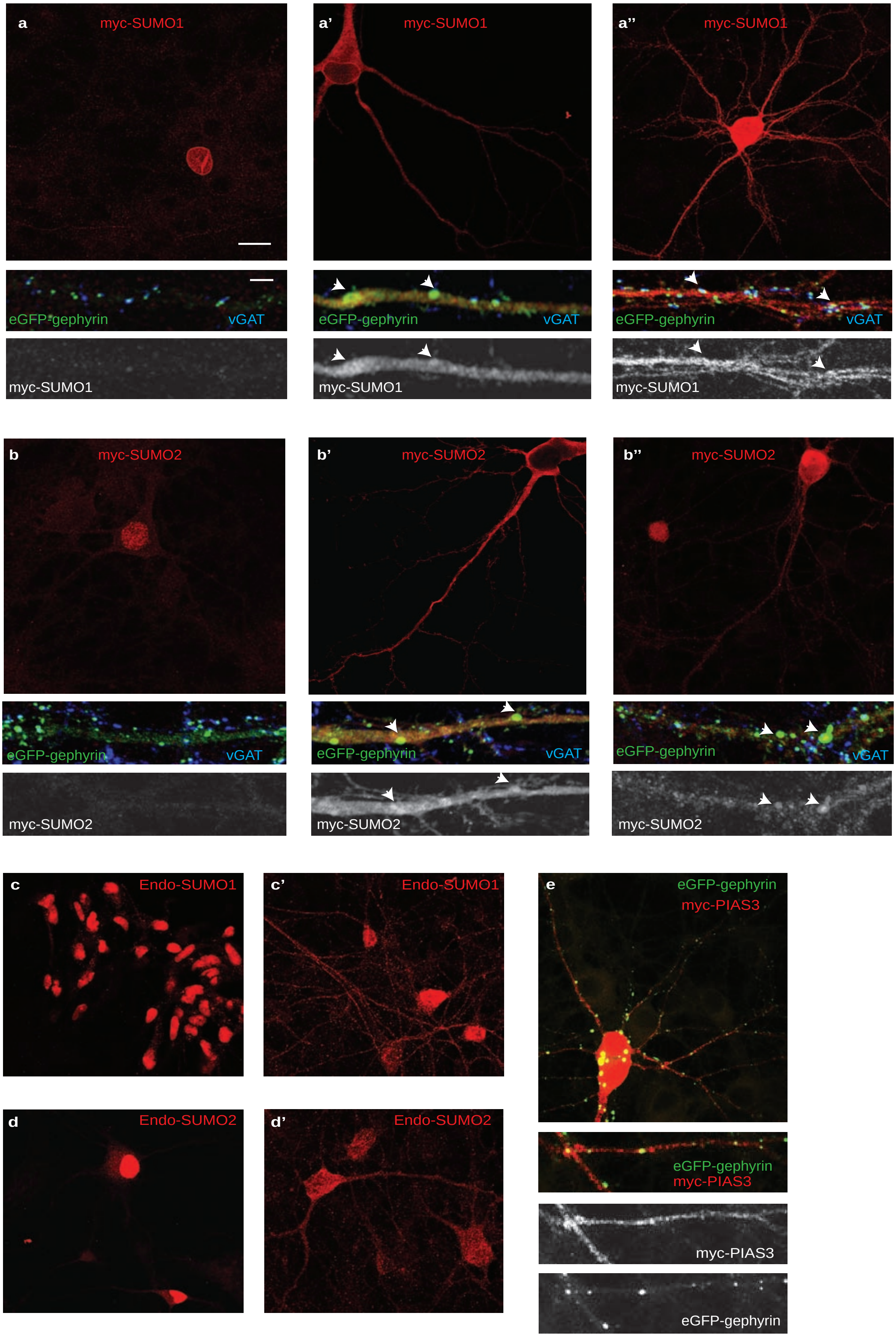

Suppl. Fig. 3: Co-expression of myc-SUMO with eGFP-gephyrin in primary neurons (DIV 8+7) (a-b'') myc-SUMO1 or myc-SUMO2 transfected neurons show three distinct subcellular localizations. myc-SUMO expression in neurons is co-localized with eGFP-gephyrin at VGAT positive terminals. Bottom panels, arrowheads show co-labelling. (c-d') endogenous SUMO1 or SUMO2 staining using commercial antibodies show nuclear, soma and dendritic staining consistent with overexpression. (e) myc-PIAS3 colocalization with eGFP-gephyrin in neuronal dendrites. Scale bars 10μm, 5μm.

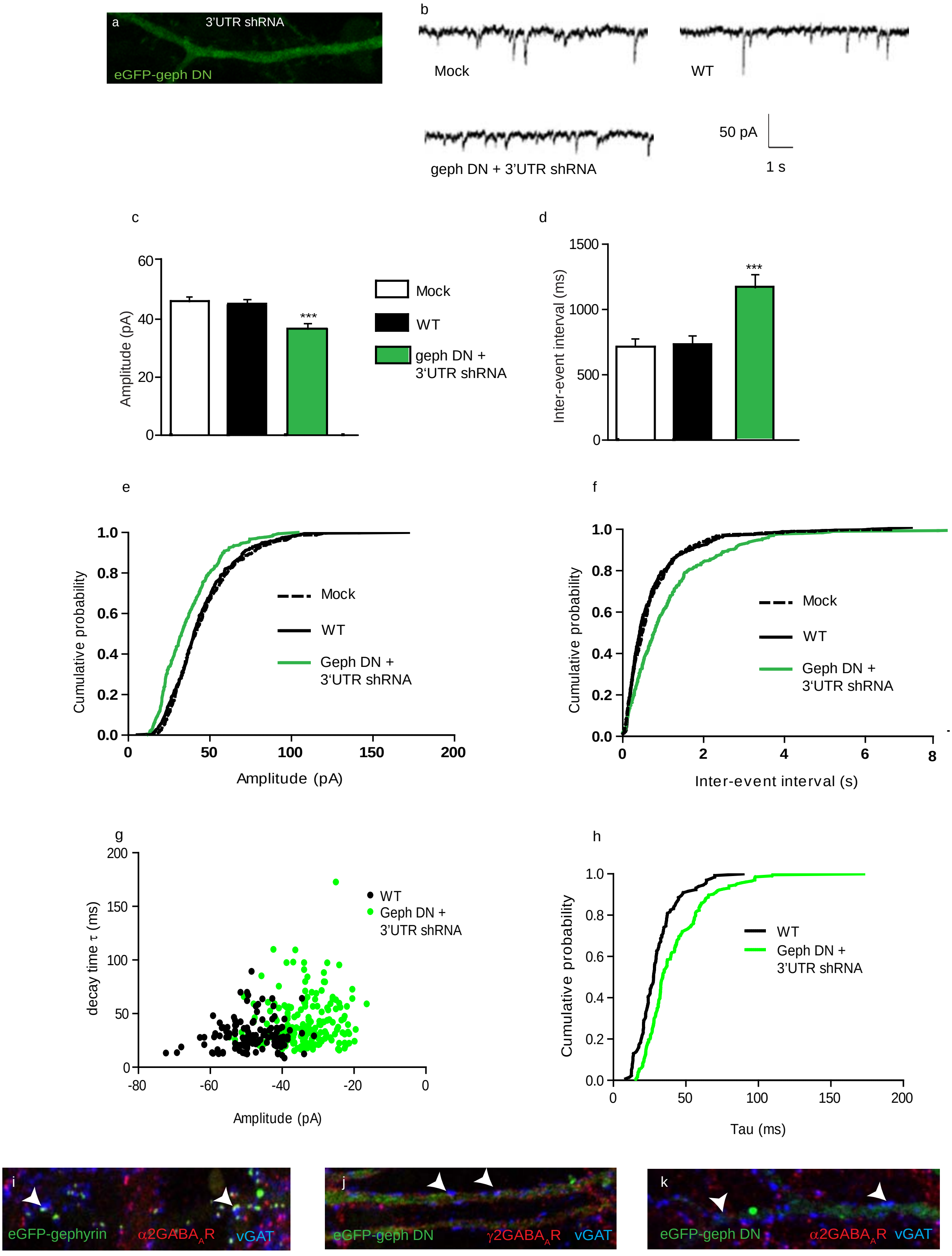

Suppl. Fig. 4: Functional analysis of primary neurons transfected with gephyrin dominant negative (DN) mutant. (a) morphology of eGFP-gephyrin DN mutatnt showing diffuse eGFP labeling along dendrites. (b) mIPSC traces in neurons transfected with eGFP-gephyrin, eGFP-gephyrin DN along with 3'UTR shRNA or mock transfected. (c) mIPSC amplitude in neurons co-transfected with either eGFP-gephyrin, eGFP-gephyrin DN along with gephyrin 3'UTR shRNA or mock transfected. (d) mIPSC inter-event interval in neurons co-transfected with eGFP-gephyrin or eGFP-gephyrin DN along with gephyrin 3'UTR ShRNA or mock transfected. (e) Cumulative probability distribution of mIPSC amplitude shows significantly reduced currents in eGFP-gephyrin DN transfected neurons. (f) Cumulative probability distribution of mIPSC inter-event interval is significantly increased in neurons transfected with eGFP-gephyrin DN. (g) Tau decay kinetics of neurons transfected with eGFP-gephyrin DN mutant is reduced compared to eGFP-gephyrin transfected neurons. (h) Cumulative probability distribution shows increased Tau decay in neurons expressing eGFP-gephyrin DN. (i-k) a2 or g2 GABAAR staining in neurons transfected with eGFP-gephyrin, eGFP-gephyrin DN shows normal GABAAR clusters in WT controls, but is highly reduced in mutant neurons. Lower panels, arrowhead shows reduced GABAAR staining. Scale bars 10µm and 5µm.

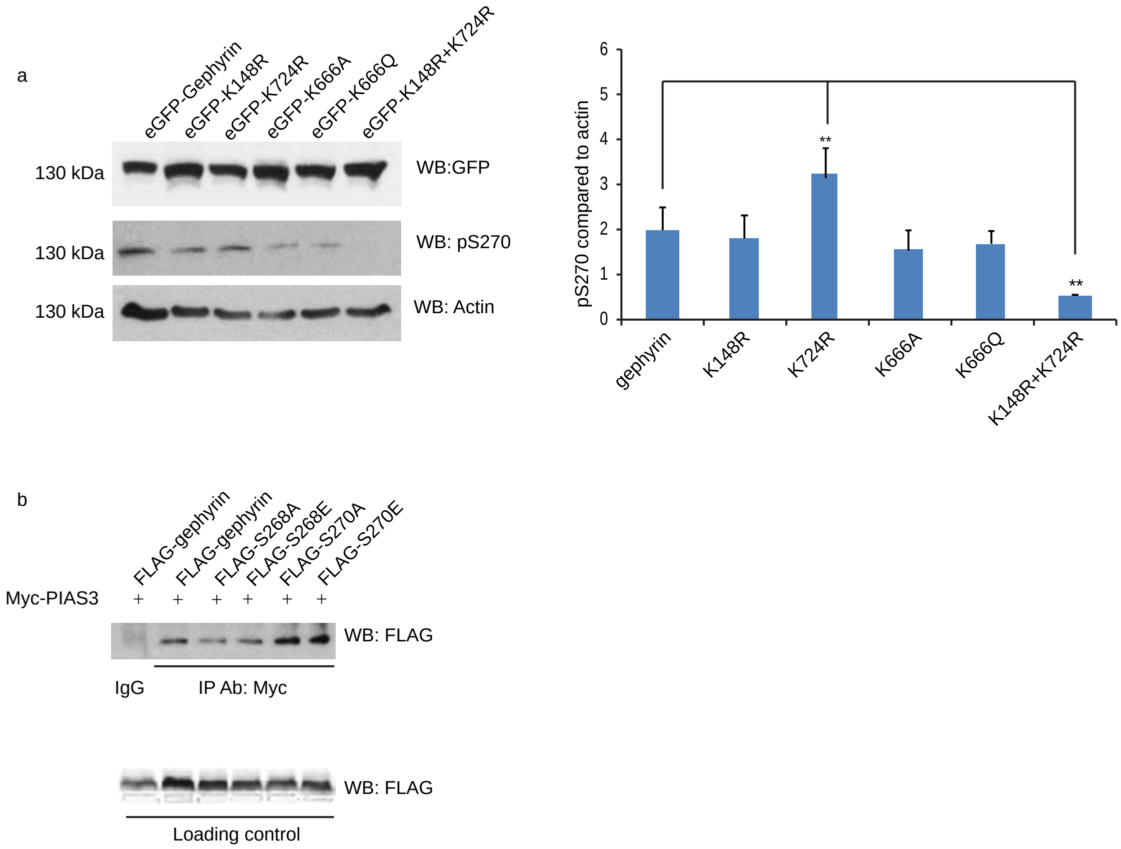

Suppl. Fig. 5: Biochemical analysis showing SUMOylation and phosphorylation are coupled in gephyrin. (a) Phospho-S270 gephyrin blot shows differences in the level of phosphorylation between different SUMO and acetylation mutations. One-way ANOVA $F(5, 16)= 3.1$, $P<0.0001$. (b) IP for myc-PIAS3 followed by WB against FLAG-gephyrin and its phospho-mutant variants show weaker binding for S268 site mutants and stronger binding for S270 site mutants.

Supplementary Table 1: Gephyrin SUMO consensus sites based on SUMOplot prediction

**Position of SUMO prediction sites**	**Score**
K666	0.93
K465	0.77
K373	0.76
K243	0.69
K602	0.63
K715	0.61
K724	0.52
K219	0.5
K328	0.2

Supplementary Table 2: Surface exposed Lys residues on gephyrin

**Position**	**SUMOplot prediction**	***In vitro* assay**	**Additional feature**
K57	no	negative	
K77	no	negative	
K101	no	negative	
K138	no	Not tested	acetylated
K148	no	SUMO1	acetylated
K149	no	Not tested	
K328	yes	negative	
K362	no	Not tested	
K497	no	negative	
K521	no	negative	
K602	yes	negative	
K666	yes	negative	acetylated
K715	yes	negative	
K724	yes	SUMO2	

Supplementary Table 3.

	Mock	WT	K148R + sh	K724R + sh	S268E/K666A sh
**Number of cells**	5	6	14	7	7
**Events**	501	531	1690	1147	305
**Amplitude**	46.24 ± 0.93	44.6 ± 0.88	59.34 ± 0.78 ***	57.7 ± 0.95 ***	36.56 ± 1.01 ***
**Inter-event Intervals (ms)**	836.3 ± 41.4	743.1 ± 43.79	503 ± 14 ***§	362 ± 10 ***§	1165 ± 88 ***

Values are given as mean ± SEM. Statistical analysis was done using one-way ANOVA, and Bonferroni's Multiple Comparison *Post-Hoc* Test. *** $p < 0.001$ between K148R+sh, K724R+sh, S268E/K666A+sh and WT; §$p < 0.001$ between K148R+sh and K724R+sh.
